# Liquid application dosing alters the physiology of air-liquid interface (ALI) primary human bronchial epithelial cell/lung fibroblast co-cultures and *in vitro* testing relevant endpoints

**DOI:** 10.3389/ftox.2023.1264331

**Published:** 2024-01-23

**Authors:** Nicholas M. Mallek, Elizabeth M. Martin, Lisa A. Dailey, Shaun D. McCullough

**Affiliations:** ^1^ Curriculum in Toxicology and Environmental Medicine, University of North Carolina, Chapel Hill, NC, United States; ^2^ Epigenetics and Stem Cell Biology Laboratory, National Institute of Environmental Health Sciences, National Institutes of Health, Department of Health and Human Services, Durham, NC, United States; ^3^ Public Health and Integrated Toxicology Division, Center for Public Health and Environmental Assessment, United States Environmental Protection Agency, Chapel Hill, NC, United States; ^4^ Exposure and Protection, RTI International, Durham, NC, United States

**Keywords:** inhalation, IVIVE, new approach methodologies (NAMs), air-liquid interface (ALI), epithelial, risk assessment, bronchial, respiratory tract

## Abstract

Differentiated primary human bronchial epithelial cell (dpHBEC) cultures grown under air-liquid interface (ALI) conditions exhibit key features of the human respiratory tract and are thus critical for respiratory research as well as efficacy and toxicity testing of inhaled substances (*e.g.*, consumer products, industrial chemicals, and pharmaceuticals). Many inhalable substances (*e.g.*, particles, aerosols, hydrophobic substances, reactive substances) have physiochemical properties that challenge their evaluation under ALI conditions *in vitro*. Evaluation of the effects of these methodologically challenging chemicals (MCCs) *in vitro* is typically conducted by “liquid application,” involving the direct application of a solution containing the test substance to the apical, air-exposed surface of dpHBEC-ALI cultures. We report that the application of liquid to the apical surface of a dpHBEC-ALI co-culture model results in significant reprogramming of the dpHBEC transcriptome and biological pathway activity, alternative regulation of cellular signaling pathways, increased secretion of pro-inflammatory cytokines and growth factors, and decreased epithelial barrier integrity. Given the prevalence of liquid application in the delivery of test substances to ALI systems, understanding its effects provides critical infrastructure for the use of *in vitro* systems in respiratory research as well as in the safety and efficacy testing of inhalable substances.

## Introduction

Exposure to a broad range of inhalable substances including inhaled pharmaceuticals, consumer products, industrial chemicals, and ambient air pollutants influences human health worldwide. Inhalation is one of the three primary routes of chemical and pharmaceutical exposure and many inhaled substances elicit their effects, beneficial or adverse, in the respiratory tract, which serves as the portal of entry. The vast number of data-poor new and existing inhalable substances, including ambient or occupational exposure-related chemicals/substances, pharmaceuticals, and consumer and commercial products, as well as mixtures of different substances, and repeated exposure scenarios precludes the feasibility of using traditional *in vivo* animal studies to evaluate the potential adverse effects of these exposures and scenarios (National Research Council, 2007). The 2007 National Research Council report, *Toxicity Testing in the 21*st *Century: A Vision and a Strategy*, outlined a strategy to address these limitations through the use of *in vitro* and computational new approach methodologies (NAMs) to advance the throughput and human relevance of toxicity testing. These recommendations have been used by regulatory agencies, including the US Environmental Protection Agency, to develop strategic plans to increase the use of NAMs to generate data for decision making (United States Environmental Protection Agency, 2020; US Environmental Protection Agency, 2021).

The respiratory tract epithelium functions as a physical barrier between inhaled materials and underlying tissues. In the nasal and tracheobronchial airways, the secretion of mucus and presence of beating cilia also function as a mucociliary escalator to facilitate clearance of inhaled materials and pathogens. Further, respiratory epithelial cells participate in the induction and regulation of the immune response to injury and inhaled insults through the secretion of pro-inflammatory cytokines (Reviewed in Polito and Proud, 1998; Hiemstra, McCray, and Bals, 2015; Hewitt and Lloyd, 2021). Primary human airway epithelial cell cultures that have been differentiated and subsequently maintained under ALI conditions exhibit key features of their respective regions of the human respiratory tract *in vivo* including the formation of a selectively permeable and electrically resistant barrier, the presence of beating cilia, release of pro-inflammatory cytokines, and secretion of mucus (Fulcher et al., 2005; Ross et al., 2007; Pezzulo et al., 2011; Randell et al., 2011; Rayner et al., 2019). ALI-differentiated primary human bronchial epithelial cell (dpHBEC-ALI) and primary human nasal epithelial cell (dpHNEC-ALI) systems have been widely used to model their respective regions of the respiratory tract to advance our understanding of respiratory tract development and disease. They are also a critical resource in support of efforts to increase the *in vivo* human relevance of inhaled test substance evaluation (Clippinger et al., 2018a; Clippinger et al., 2018b; Faber and McCullough, 2018; Hiemstra et al., 2018).

Evaluating the effects of inhaled gases and vapors on differentiated primary airway epithelial cell culture models while maintaining ALI conditions can generally be accomplished using existing *in vitro* exposure apparatus/technology. In contrast, many other types of test substances (*e.g.*, particles, aerosols, hydrophobic substances, reactive substances, biomolecules, *et cetera*) have physical and/or chemical properties that pose challenges to their evaluation under ALI conditions (examples reviewed in Lacroix et al., 2018). The evaluation of these inhaled methodologically challenging chemicals (MCCs) using *in vitro* systems has often relied on the application of test substances in aqueous solutions/suspensions to the apical surface of ALI models of the respiratory tract (described here as “liquid application”, but also commonly referred to as “direct application” or “liquid dosing”). Liquid application dosing is also often used to deliver exogenous stimuli (*e.g.*, lipopolysaccharide (LPS), tumor necrosis factor alpha (TNF-a), or small molecules to ALI cultures for respiratory biology research and pharmaceutical development, respectively.

When using liquid application dosing, the effect of the test substance treatment is typically assessed by comparison to the respective aqueous solvent (*e.g.*, water, saline, buffered saline, cell culture medium, *et cetera*) as a vehicle control. The application of exogenous liquid alone (*i.e.*, in the absence of a test substance) disrupts ALI conditions, a key physiologically relevant feature of these culture models; however, the effects of applying liquid to differentiated ALI cultures are typically not considered in the analysis and interpretation of study data. Maintaining ALI conditions is key for the successful differentiation of pHBECs into dpHBECs as ALI increases the available oxygen at the epithelial cell layer, which is critical for successful cell differentiation; and the loss of ALI conditions by exogenous liquid application reduces oxygen availability and may alter dpHBEC characteristics (Kouthouridis et al., 2021). Further, while liquid application dosing is commonly used for the delivery of test substances and biological stimuli in ALI systems in inhalation toxicology research and testing, the effects of applying liquid to ALI cultures have not been well characterized. Thus, the potential for liquid application dosing to affect the physiological relevance of the differentiated ALI *in vitro* system, as well as confound test substance exposure effects and the extrapolation of study data to human health outcomes are poorly understood. Addressing this knowledge gap by determining whether disrupting the *in vivo*-relevant ALI conditions alters dpHBEC culture physiology is critical to providing context for the use of liquid application dosing of *in vitro* ALI systems for respiratory biology, inhalation toxicology, pharmaceutical development, and disease research. Here, we report the results of a study describing the effects of the application of liquid, in the absence of a test substance, to a dpHBEC-ALI co-culture model on endpoints that are relevant to *in vivo* bronchial epithelial tissue physiology. These endpoints included global transcriptional regulation, biological pathway activity, ciliary beat frequency, regulation of stress-responsive cellular signaling pathways, release of pro-inflammatory cytokines and growth factors, and epithelial barrier function.

## Methods

### Differentiated dpHBEC/HLF co-culture ALI model

Fibroblasts exist immediately beneath the bronchial epithelial barrier and contribute to respiratory tract homeostasis and disease (Kendall and Feghali-Bostwick, 2014; White, 2015). We have also recently observed that lung fibroblasts are both a target and mediator of the effects of inhaled toxicant exposure *in vitro* (Faber et al., 2000). Thus, we utilized a modified version of the co-culture model used by Faber *et al.* (2000) that combined dpHBEC on cell cultures inserts at ALI with confluent monolayers of IMR90 lung fibroblasts. The use of this system allowed for an improved relevance to the *in vivo* tissue compared to the use of dpHBEC cultures alone while also allowing for the evaluation of cell type-specific effects of liquid application.

**FIGURE 1:**
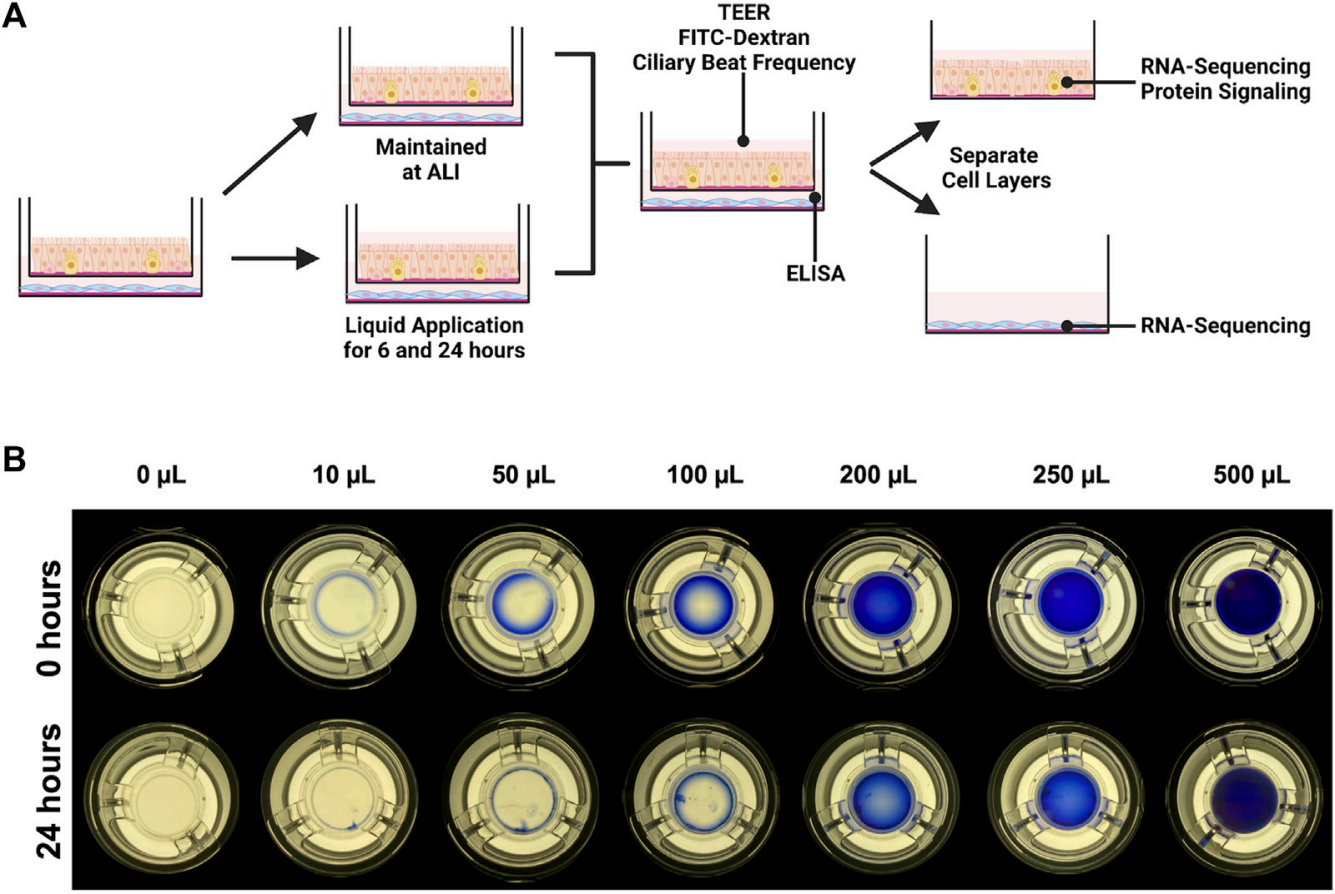
Experimental design. **(A)** IMR90 fibroblasts were seeded onto collagen-coated tissue culture wells 24 h before being combined with dpHBEC under ALI conditions. 24 h after the cells were combined, 250 µL of liquid (ALI medium) was applied to the apical surface of the dpHBECs for either 6 or 24 h. A matched set of cultures was maintained under ALI conditions to serve as the control. The indicated endpoints were evaluated to determine the effect of the application of liquid on dpHBEC cultures. **(B)** Titration of apical volumes of crystal violet solution in 12 mm Transwell^®^ inserts used to visualize the relative coverage of the insert surface area at different applied liquid volumes. The 250 µL volume was determined to provide the best balance between the smallest volume and uniformity of liquid coverage. **(A)** created with BioRender.com.

pHBEC were obtained via bronchial brushing from healthy donors of 18–40 years of age that were not currently smoking and had no more than a one pack-year of lifetime smoking history. Donors gave their consent after being informed of risks and procedures. The consent and collection protocol were approved by the UNC School of Medicine Committee on the Protection of the Rights of Human Subjects and by the US EPA. Collection of pHBEC from volunteers was performed in accordance with relevant guidelines and regulations. pHBEC were isolated from brush biopsy (“passage 0”) and expanded to passage 3 prior to being plated on 12 mm Transwell^®^ inserts (Corning #3460; 0.4 µm pore polyester membranes), becoming confluent, and differentiating for 24 days under ALI conditions. Detailed descriptions of the techniques, reagents, and materials used for the culture/expansion of pHBEC and differentiation of pHBEC at ALI used in this study are available as open access methods document by Dailey and McCullough (Dailey and McCullough, 2021). Day 24 ALI cultures were visually evaluated for the presence of beating cilia and the production of mucus as indicators of differentiation status to qualify their use in experiments. Donor demographics are listed in Supplementary Table S1. All cultures were at ALI day 24 at the beginning of experiments. The human lung fibroblast cell line IMR90 (Nichols et al., 1977) was obtained from the American Type Culture Collection (ATCC, No. CCL-186, Batch #64155514), and cultured as described in detail in the open-access methods document by Mallek and McCullough (Mallek and McCullough, 2021a). Short tandem repeat (STR) service provided by ATCC (cat #135-XV) was used to authenticate IMR90 cells (Supplementary Figure S1). As described in Figure 1A, IMR90 fibroblasts were seeded onto a collagen-coated 12-well plate (Corning, #3512) at a density of 1 × 10^5^ cells/mL in 800 µL of Pneumacult ALI medium (StemCell Technologies, #05001). The following day, day 23 dpHBEC-ALI inserts were added into the fibroblast-seeded wells (dpHBEC-IMR90 ALI model). The dpHBEC-IMR90 ALI model was used for experiments on the following day. The dpHBEC-IMR90 ALI model was maintained at ALI or subjected to the addition of ALI medium to the apical surface of the culture (“liquid application”). The methods for the selection of the volume of medium applied are described in the following section. The cells were kept submerged in ALI medium for either 6 or 24 h to represent “early” and “late” effects, respectively. The 24-h treatment is consistent with the liquid application treatment duration utilized in the recent OECD case study on the use of an ALI-differentiated *in vitro* NAM for an IATA to refine inhalation risk assessment for point of contact toxicity (Vinall, 2017; Hiemstra et al., 2018; Paulo, 2020; Welch et al., 2021). The effect of liquid application on global gene expression, stress-responsive signaling protein phosphorylation, growth factor secretion, pro-inflammatory cytokine secretion, trans-epithelial electrical resistance (TEER), and permeability to a 20 kDa fluorescent dextran was then determined immediately after 6 and 24 h of liquid application (Figure 1A). A set of *n* = 3 donors were used for RNA-Sequencing, differential gene expression analysis, TEER, and FITC-dextran permeability. Follow-up experiments including immunoblotting and ELISAs were performed with an additional *n* = 3 donor set, unique from the donors used in initial experiments.

### Determination of liquid application volume

We were not able to find any published studies evaluating the completeness of culture coverage resulting from different applied liquid volumes. The development of discontinuous coverage of the culture surface by the applied liquid would be expected to result in in consistencies in test substance concentration and flux, ion and/or nutrient concentrations, and exposed oxygen concentrations across the culture. Thus, we sought to determine the smallest applied liquid volume that resulted in complete, and most uniform, coverage of the cell layer during a 24-h treatment period. To accomplish this, we titrated a range of apical volumes (10 μL–500 μL) of a 1:10 Trypan Blue (Sigma, #T8154-20 ML) solution in Dulbecco’s Phosphate Buffered Saline (Gibco #14190-144)) to the apical surface of dpHBEC-ALI cell layers grown on 12 mm Transwell^®^ inserts (Figure 1B). After the 24-h treatment duration, inserts were placed on a white light transilluminator (FUJIFILM #IPE4046) and photographed with an iPhone SE (Apple, Model #MX9M2LL/A). The lowest volume that maintained complete coverage of the cell layer during the 24-h treatment duration with the best uniformity was selected for use in the study.

### RNA isolation

Total RNA was isolated using the PureLink RNA Mini kit (ThermoFisher #12183025) according to the detailed open access method by McNabb and McCullough (McNabb and McCullough, 2020). Briefly, dpHBEC samples were harvested by the addition of 250 µL RNA lysis buffer containing 1% β-mercaptoethanol, triturated, and transferred to Eppendorf tubes on ice prior to storage at −80°C until extraction. Total RNA was eluted in nuclease-free water (ThermoFisher, #AM9937) following extraction and column purification. Total RNA was quantified using a NanoDrop OneC UV-Vis Spectrophotometer. Purified RNA was stored at −80°C until submission for RNA-sequencing or preparation of cDNA for quantitative real-time polymerase chain reaction (qRT-PCR).

### Sequencing, DESeq2, and qRT-PCR validation

Library preparation and sequencing were performed by the University of North Carolina at Chapel Hill High Throughput Genomic Sequencing Facility. Briefly, 100–500 ng of RNA were used to make libraries using a KAPA Stranded mRNA-Seq Kit, with KAPA mRNA Capture Beads (Roche Sequencing and Life Science #07962207001). Libraries underwent quality control via dsDNA High Sensitivity Qubit following cDNA synthesis and adaptor ligation (Q32851). Libraries were normalized to 5 nM and pooled in equal amounts and then processed on a miSeq nano flowcell (MS-103–1001). Samples were run on an Illumina Novaseq 6,000 (Illumina, San Diego, CA), and calibrated against 1% PhiX as a positive control during sequencing. Base call files were transformed into FASTQ files using bcl2fastq software (version 2.20.0). Mapping of sequence reads to the human genome (GRCh38) was performed by STAR (version 2.5) with the following parameters: - outSAMattrIHstart 0 --outFilterType BySJout--alignSJoverhangMin 8 -- outMultimapperOrder Random (other parameters at default settings). Mapped read counts per gene were collected by Subread feature Counts (version 1.5.0-0-p1). Genes with a minimum average of normalized mapped read counts >50 in at least one category were selected for differential gene expression analysis. Differentially expressed genes (DEGs) were identified by DESeq2 (v 3.13) using filters of FDR<0.01. Read counts and the processed data matrix are available to the public on the NCBI Gene Expression Omnibus (series number: GSE198884). DEG datasets were also generated for the underlying IMR90 fibroblasts after the application of liquid to the apical dpHBECs for either 6 or 24 h (Supplementary Figure S2). Fold change expression values derived from RNA-sequencing-derived DESeq2 were validated against standard real-time qRT-PCR methods (Supplementary Figure S4). Briefly, cDNA was synthesized using the iScript Reverse Transcription Kit (Bio-Rad, #1708891) according to the manufacturer’s protocol. Fold change values were assessed via primers, hydrolysis probes (Supplementary Table S2), and iTaq universal Probes Supermix (Bio-Rad, #1725131) on a CFX96 Touch (Bio-Rad). Target gene C_q_ values were normalized to β-actin and expressed as fold changes between liquid application and ALI conditions using the Pfaffl method (Pfaffl, 2001).

### Ingenuity Pathway Analysis

The number of statistically significant alternatively regulated genes identified in dpHBECs following 24-h liquid application treatment (10,239 genes) exceeded the maximum number of genes (8,000) that can be analyzed by the IPA software. Thus, we generated a sub-set of each DEG list that included only statistically significant genes that were alternatively regulated with an absolute value log_2_ fold change >2.0. These “pathway analysis” gene sets were uploaded to Ingenuity Pathway Analysis software (Version 65367011). Gene lists were exported for each of the top 10 canonical pathways as determined by -log (*p*-value). The 10 genes with the greatest absolute fold change value were exported as tables for the top 10 most significant canonical pathways. IPA was also used to determine canonical pathways and top genes for underlying IMR90s after liquid application to apical dpHBECs (Supplementary Figure S3, 4).

### Protein collection and immunoblotting

Total protein was isolated via a RIPA extraction. Briefly, dpHBEC samples were harvested by the addition of 250 µL of RIPA lysis buffer (50 mM tris-base, pH 8.0, 150 mM NaCl, 400 uM EDTA, 10% glycerol, 1% Triton-X, 0.1% SDS, 0.1% sodium deoxycholate, with 1X protease inhibitor (Sigma, #11697498001) and phosphatase inhibitor (Sigma, #04906837001) cocktails) to each insert, scraping with a wide-bore P200 pipette tip (United States Scientific, #10118410), and transfer to an Eppendorf tube prior to incubation on ice for 20 min. Samples were centrifuged at 13,000 × *g* for 15 min to pellet cell debris and supernatants were transferred to new Eppendorf tubes on ice. Three aliquots (10 µL) were removed from each sample for determination of protein concentration by BCA assay. 5X Laemmli buffer (120 mM Tris, pH 6.8, 400 mM dithiothreitol, 20% glycerol, 4% SDS, 0.025% bromophenol blue) was then added to the remaining sample volume before the samples were incubated at 95 °C for 5 min. Samples were stored at −80°C until used for immunoblotting. The antibodies and dilutions used for immunoblotting are provided in Supplementary Table S3. All samples were electrotransferred on 0.45 µM nitrocellulose membranes (Bio-Rad, #1620115) in transfer buffer (48 mM tris-base, 39 mM glycine, 20% methanol). Chemiluminescence was generated via a horseradish peroxidase-conjugated secondary antibody (Jackson ImmunoResearch, #711-036–152) and Clarity Western ECL Blotting Substrate (Bio-Rad #1705060). Images were taken on a ChemiDoc MP Imaging System (Bio-Rad), and densitometry was generated using Bio-Rad Image Lab Software (Version 6.1.0, Build 7).

### Evaluation of growth factor and pro-inflammatory cytokine secretion

Conditioned medium was collected from the basolateral compartment of dpHBEC-ALI cultures immediately before liquid application, and 6 or 24 h after liquid application. Conditioned media were centrifuged and syringe filtered through 0.22 µm pore PVDF membranes (Millipore Sigma, #SLGV033RS) to remove cellular debris before storage at −80°C until use. Conditioned media were probed for cytokine secretion by the V-PLEX Human Proinflammatory Panel II (4-Plex) (Mesoscale Discovery, K15053D-1) and growth factor secretion by the U-PLEX Development Pack (Mesoscale Discovery, K15228N-1) according to the manufacturer’s protocols. Briefly, conditioned media samples were diluted two-fold, and assayed in triplicate with either four 10-fold serial dilutions (for cytokines) or four two-fold serial dilutions (for growth factors) to determine the limits of detection in our dpHBEC-ALI cultures. The MSD plate was washed thrice with 150 µL of wash buffer per well prior to the addition of 50 µL of diluted sample to individual wells and incubation with shaking (300 RPM) for 2 hours at room temperature. Samples were then aspirated and the plate was washed thrice with 150 µL of wash buffer prior to the addition of 25 µL of detection antibody solution to each well and incubation with shaking (300 RPM) for 2 hours at room temperature. The antibody solution was then aspirated and the plate was washed thrice with 150 µL wash buffer before the addition of 150 µL of 2X Read Buffer T to each well. The sample plate was read on the Meso Quickplex SQ 120, and analyte concentrations were determined using the MSD Discovery Workbench software (Version 4.0.12)

### Trans-epithelial electrical resistance

Trans-epithelial electrical resistance (TEER) was measured using the EVOM2 (World Precision Instruments) with EndOhm cup for 12 mm inserts (World Precision Instruments) at the indicated time points as described in a detailed open-access methods document by Mallek and McCullough (Mallek and McCullough, 2021b). Briefly, resistance measurements were obtained for both experimental samples and insert only (without cells). TEER values from insert only samples were subtracted from experimental sample resistance values and the resulting values were then multiplied by the insert surface area to calculate values in Ω × cm^2^ (Srinivasan et al., 2015). Three cultures were used for each condition per experiment and each culture was assayed in technical triplicate. The mean ( ± SD) of three independent experiments is shown. TEER was treated as a destructive assay and no additional assays were conducted on cultures that were analyzed by TEER.

### Fluorescein isothiocyanate-labeled dextran assay

The translocation of a 20 kDa fluorescein isothiocyanate (FITC)-labeled dextran (Sigma, FD20) was measured as described in a detailed open-access methods document by Faber and McCullough (Faber and McCullough, 2020). Briefly, experimental sample inserts and insert only (without cells) were transferred to a new multi-well plate containing 800 µL of phenol-red free MEM (ThermoFisher, #51200038) in each well. The apical medium was carefully aspirated from cultures after liquid application for 6 or 24 h and 250 µL of a 1 mg/mL suspension of FITC-dextran in Pneumacult ALI medium (StemCell Technologies, #05001) was added to the apical compartment of each insert. Samples were incubated in the dark for 20 min at 37°C, and the basolateral media containing translocated FITC-dextran suspension was collected and transferred to a 96-well clear bottom microplate (Corning, #3904) prior to determination of the fluorescence of each sample at 490/520 nm Ex/Em on a ClarioStar Plus plate reader. Three cultures were used for each condition per experiment and each culture was assayed in technical triplicate. The mean ( ± SD) of three independent experiments is shown. The FITC-dextran assay was considered to be destructive and no additional assays/endpoints were conducted/evaluated on cultures after this assay.

### Statistical analysis

Statistical analyses for protein densitometry, cytokine and growth factor secretion, TEER, FITC-dextran, ciliary beat frequency and total ciliated area, and viability were conducted using GraphPad Prism 9.0.1 (GraphPad Software). The statistical significance of differences between each duration of liquid application and untreated controls were evaluated using a parametric unpaired *t*-test. A *p*-value of ≤ 0.05 was considered statistically significant for all analyses.

## Results

### Applied volume affects the completeness of culture exposure

There are a broad range of applied liquid volumes used for the delivery of experimental treatments to ALI cultures reported in the literature; however, we were not able to identify any published studies that evaluated the completeness of culture coverage at the beginning or end of experiments utilizing liquid application dosing. Thus, we sought to empirically determine the smallest volume that maintained the most complete coverage of the culture over the 24-h treatment. The application of 200 μL (177 μL/cm^2^) provided complete coverage immediately after liquid application; however, coverage became markedly uneven during the 24-h treatment duration (Figure 1B). In contrast, the application of 250 μL (221 μL/cm^2^, equivalent to the addition of 73.5 µL to a 6.5 mm cell culture insert) was the smallest volume tested that maintained complete coverage across the insert, while also exhibiting better uniformity than the 200 μL volume, by 24 h. Thus, we utilized an applied liquid volume of 250 μL per 12 mm cell culture insert for subsequent experiments. The application of lower volumes (*i.e.*, ≤ 100 μL; ≤ 88 μL/cm^2^, equivalent to ≤ 30 µL applied to a 6.5 mm cell culture insert) failed to completely cover the culture surface at the time of application. The completeness of culture coverage at these volumes was diminished over the 24-h incubation period. Importantly, there is no way to achieve completely uniform depth across the surface area of the insert due to the formation of a meniscus.

### Liquid application alternatively regulates global gene expression in dpHBEC cultures

**FIGURE 2:**
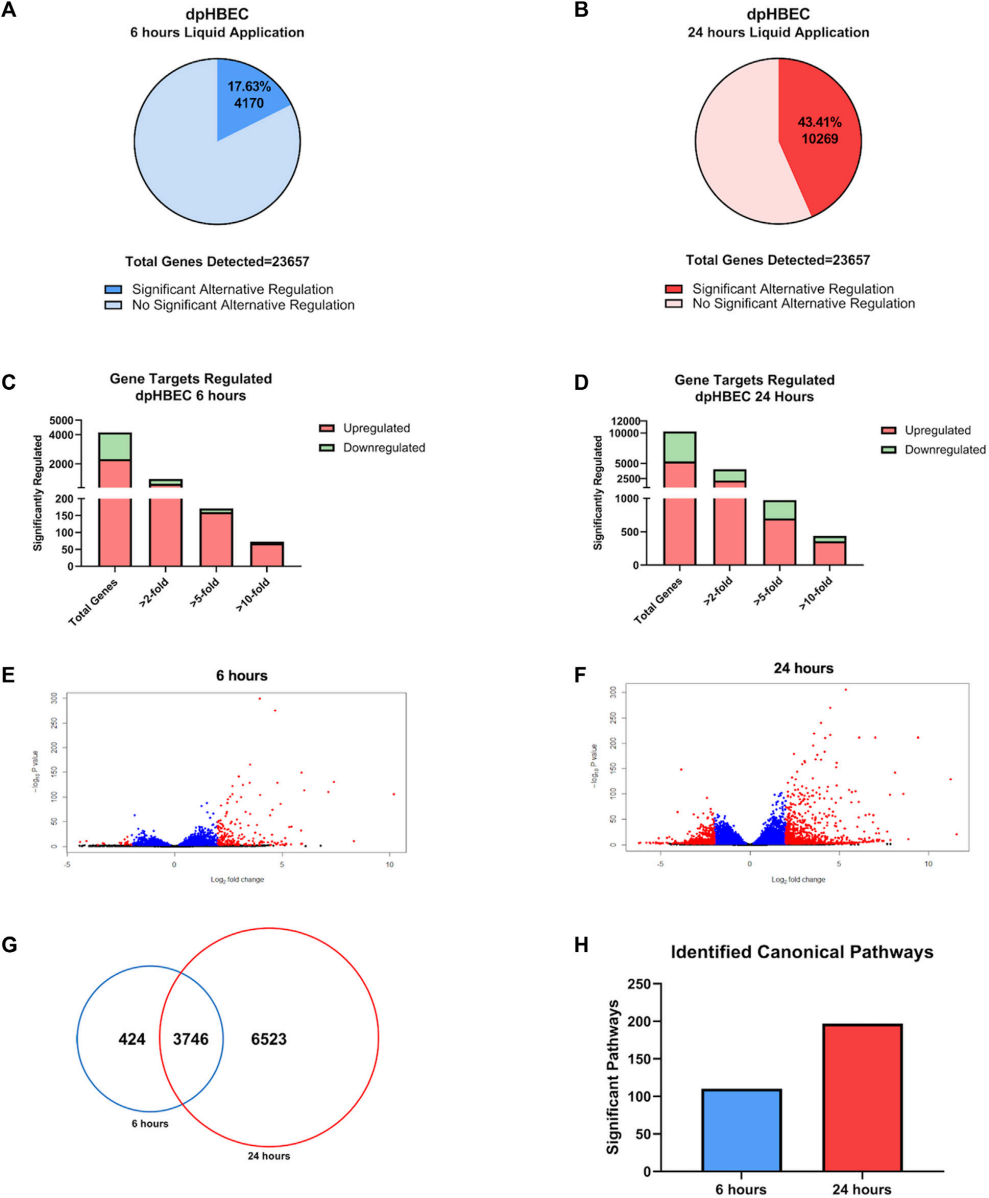
Global transcriptome changes following liquid application exposure for 6 or 24 h **(A, B)** The total amount of significantly alternatively regulated genes (adjusted *p*-value < 0.05) after 6 h **(A)** or 24 h **(B)** of liquid application, respectively. **(C, D)** The total number of significant alternatively regulated genes from **(A, B)** separated into fold change expression thresholds after 6 h **(C)** or 24 h **(D)** of liquid application, respectively. **(E, F)** Volcano plots of the gene datasets described in **(A, B)**. Blue points have an adjusted *p*-value of <0.01, and red points have an adjusted *p*-value of <0.05 and a log_2_ (foldchange) > 1. **(G)** Venn Diagram comparison displaying the number of significant (adjusted *p*-value < 0.05) unique and shared genes between the 6- and 24-h liquid application dataset described in **(A, B)**. **(H)** Total number of significant canonical pathways regulated after liquid application for 6 or 24 h according to Ingenuity pathway analysis of the dataset from **(A, B)**. Data shown represent *n* = 3 donors.

Following ALI differentiation, the transcriptional profiles of dpHBEC cultures recapitulate those of their corresponding *in vivo* tissue (Fulcher et al., 2005; Pezzulo et al., 2011; Randell et al., 2011). We began our assessment of the impact of liquid application on dpHBEC-ALI cultures with the evaluation of its effect on global transcriptional programming using RNA-sequencing. Of the tested transcripts (*n* = 33,121 in the RNA-seq Data set), 23,657 (71%) were detected in dpHBECs. The application of liquid to dpHBEC-ALI cultures for 6 and 24 h resulted in the significant (adjusted *p* ≤ 0.05) alternative expression of 4,170 and 10,269 genes, respectively (Figures 2A, B). Of the 4,170 genes that were alternatively expressed at 6 h of liquid application, 626 and 326 were up- and downregulated greater than 2-fold, 160 and 11 were up-and downregulated greater than 5-fold, and 68 and 5 were up- and downregulated greater than 10-fold, respectively (Figure 2C). Of the 10,269 genes that were alternatively expressed at 24 h of liquid application, 2,159 and 1,865 were up- and downregulated greater than 2-fold, 700 and 274 were up- and downregulated greater than 5-fold, and 358 and 79 were up- and downregulated greater than 10-fold, respectively (Figure 2D). The 6- and 24-h time points had 424 and 6,523 unique alternatively expressed genes, respectively, and 3,746 alternatively expressed genes in common (Figure 2G). The alternative expression of selected targets identified in the RNA-seq analysis (IL-8, IL-1α, and COX-2) were validated by qRT-PCR (Supplementary Figure S2).

**FIGURE 3:**
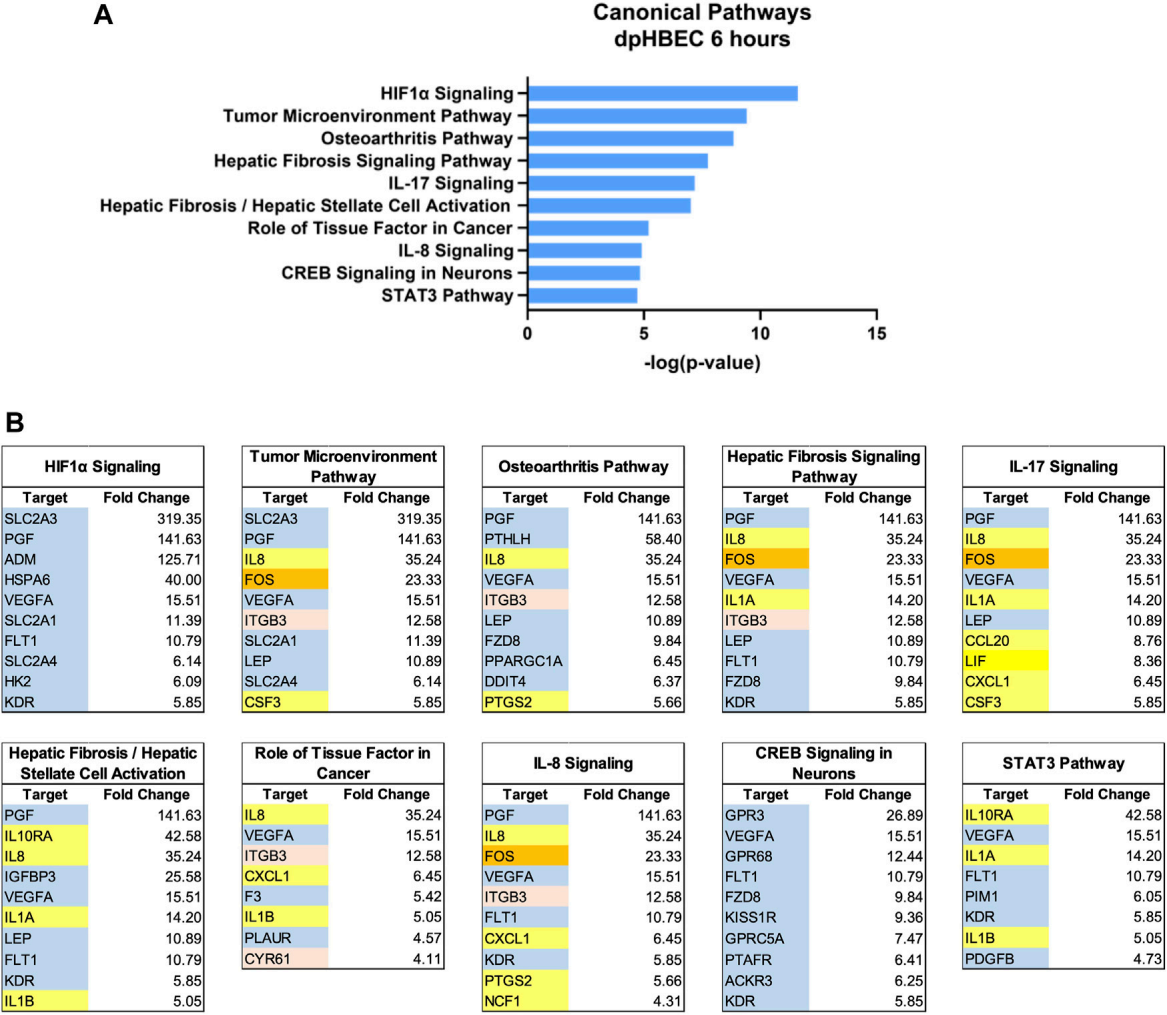
Ingenuity pathway analysis of alternatively regulated genes after 6 h of liquid application on dpHBEC-ALI cultures. **(A)** The top 10 most significant canonical pathways by the–log (*p*-value) identified after 6 h of liquid application. **(B)** The top 10 genes, by absolute value, in each canonical pathway identified in **(A)**, and their respective fold change expression over ALI cultures. Only genes exhibiting a ± log_2_ fold change ≥ 2.0 were used in this analysis. In **(B)**, blue shading indicates cell signaling-related genes, orange shading indicates transcription factors, red shading indicates adhesion-related genes, and yellow shading indicates inflammation-related genes.

**FIGURE 4:**
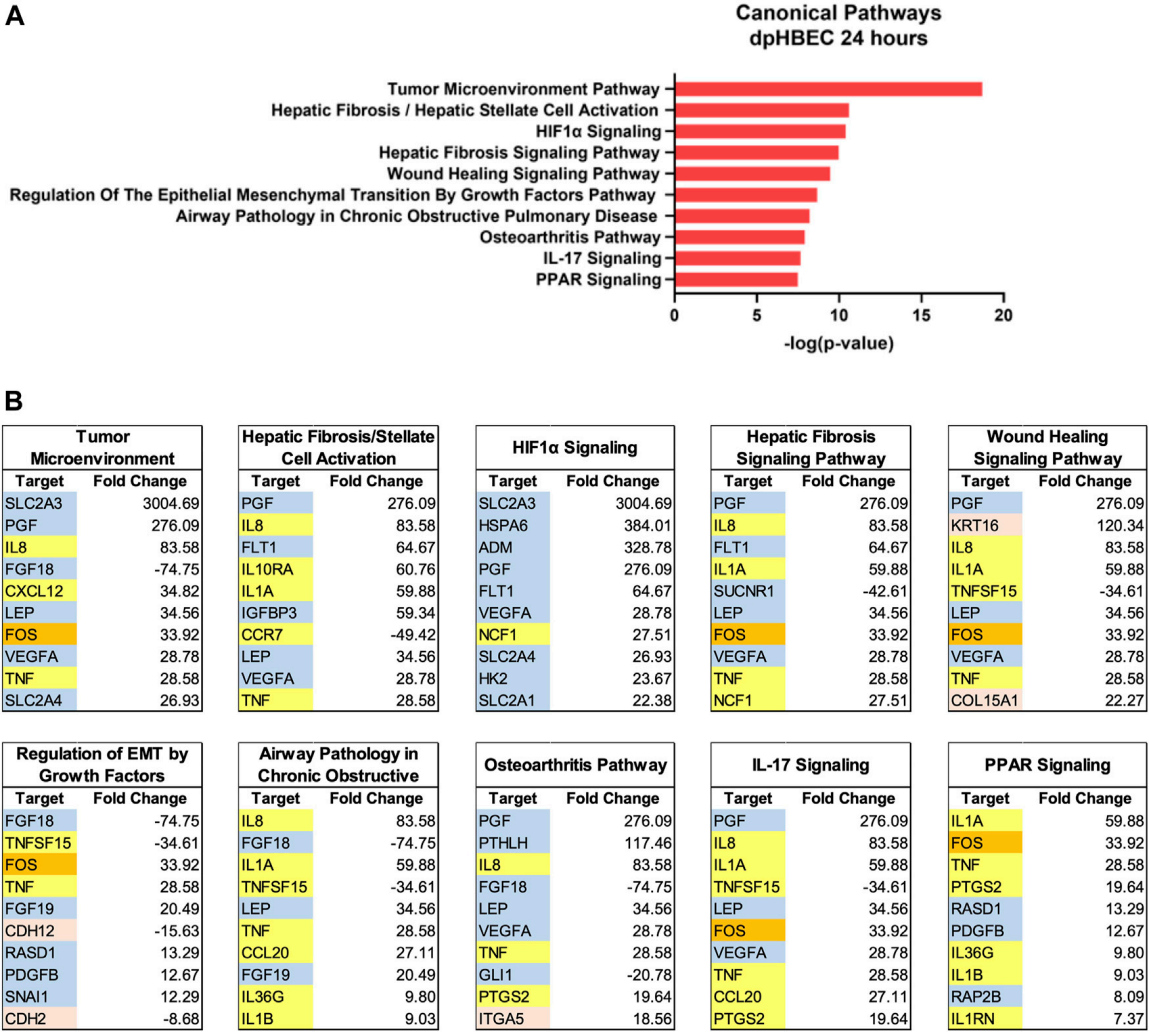
Ingenuity pathway analysis after 24 h of liquid application on dpHBEC-ALI cultures. **(A)** The top 10 most significant canonical pathways by the–log (*p*-value) identified after 24 h of liquid application. **(B)** The top 10 genes, by absolute value, in each canonical pathway identified in **(A)**, and their respective fold change expression over ALI cultures. Only genes exhibiting a ± log_2_ fold change ≥ 2.0 were used in this analysis. In **(B)**, blue shading indicates cell signaling-related genes, orange shading indicates transcription factors, red shading indicates adhesion-related genes, and yellow shading indicates inflammation-related genes.

Ingenuity Pathway Analysis of significantly alternatively expressed genes indicated that liquid application resulted in the significant alternative regulation of 110 and 197 canonical pathways in pHBEC-ALI cultures at 6 and 24 h, respectively (Figure 2H). The 10 most significant pathways (by -log (*p*-value)) identified at 6 and 24 h are listed in Figures 3A; Figure 4A and the 10 genes with the largest magnitude of fold change expression, by absolute value, for each of the top alternatively regulated pathways are listed in Figures 3B; Figure 4B, respectively. Several alternatively expressed genes were common to the 10 most significantly alternatively regulated pathways following 6 h of liquid application, including: the growth factors placental growth factor (PGF, 141.6-fold) and vascular endothelial growth factor A (VEGFA, 15.5-fold) and pro-inflammatory mediators such as interleukin (IL)-8 (IL-8, 35.2-fold), IL-1α (IL-1α, 14.2-fold), IL-1β (IL-1β, 5.1-fold), and cyclooxygenase-2 (COX-2 5.7-fold). Many alternatively expressed genes were also common to the alternatively regulated pathways after 24 h of liquid application including: the growth factors PGF (276.1-fold) and VEGFA (28.9-fold), and the pro-inflammatory mediators IL-8 (83.6-fold), IL-1α (59.9-fold), IL-1β (9.0-fold), and PTGS2 (19.6-fold). Additional highly expressed genes at 24 h included platelet derived growth factor subunit B (PDGF-B, 12.7-fold), and tumor necrosis factor (TNF, 28.6-fold).

### Liquid application increases HIF-1a protein levels and the phosphorylation of ERK and p38 cellular signaling pathways in dpHBEC cultures

**FIGURE 5:**
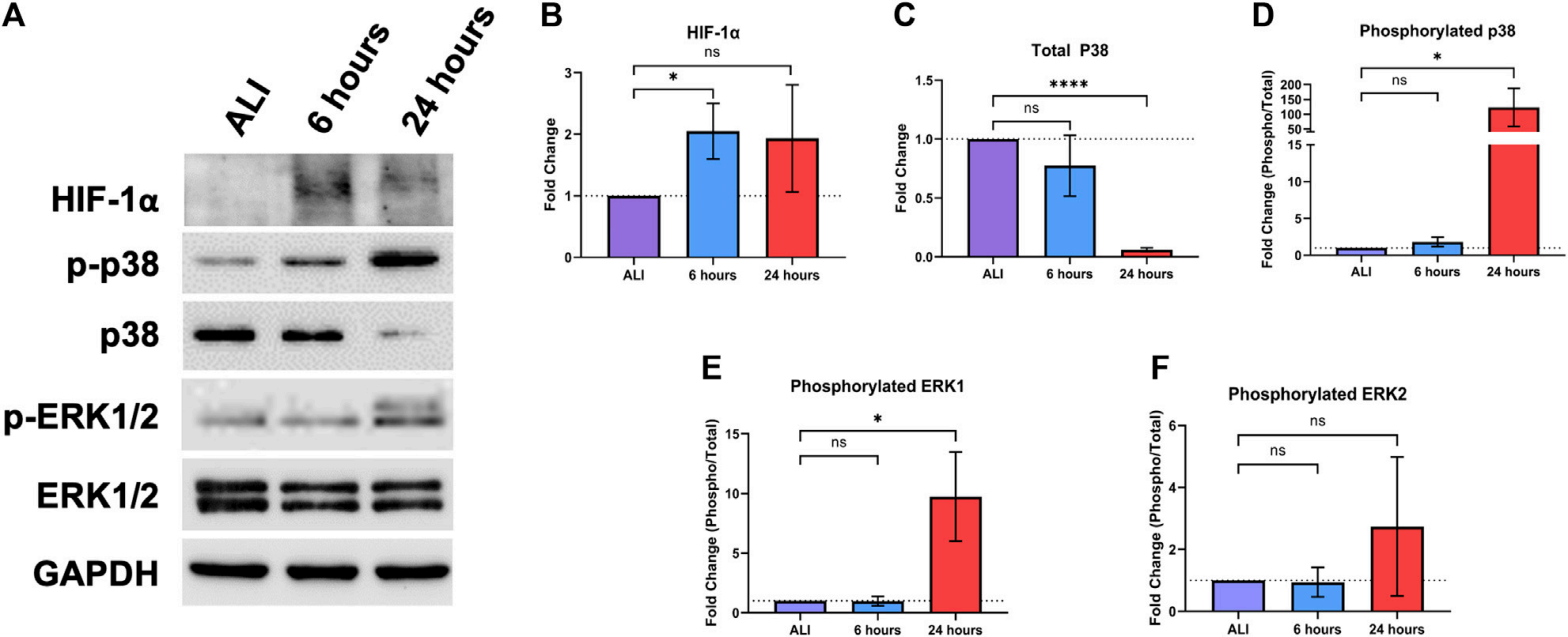
Liquid application modulates stress-responsive protein signaling pathways in dpHBECs. **(A)** Representative images of protein signaling western blots for levels of HIF-1α, phosphorylated and total p38, phosphorylated and total ERK1/2, and GAPDH loading control. Representative images represent *n* = 3 donors. Densitometry showing **(B)** changes in the induction of HIF-1α, **(C)** the ratio of phosphorylated to total p38, **(D)** total p38, **(E)** the ratio of phosphorylated to total ERK1, and **(F)** the ratio of phosphorylated to total ERK2 normalized to GAPDH loading control. Data represent the mean of *n* = 3 donors and error bars represent SD. *, ****, and ns indicate *p* ≤ 0.05, *p* ≤ 0.0001, and not significant, respectively.

HIF-1 is a heterodimeric transcription factor that regulates the expression of genes involved in the cellular response to hypoxia, as well as tissue vascularization and the maintenance of tissue homeostasis (Ziello et al., 2007; Shimoda and Semenza, 2011). HIF-1 activation is regulated by the stabilization of the HIF-1α subunit, which occurs in response to hypoxic conditions (Shimoda and Semenza, 2011). The application of liquid to an ALI culture reduces exposure of the apical culture surface to oxygen. Thus, we evaluated whether liquid application resulted in the stabilization of HIF-1α, the regulatory subunit of HIF-1, which was significantly increased (2.05-fold ( ± 0.45)) at 6, but not 24, hours of liquid application (Figure 5B).

The ERK and p38 mitogen activated protein kinase (MAPK) signaling pathways regulate the response of the respiratory epithelium to oxidative, inflammatory, and pathogenic stimuli (Roux and Blenis, 2004; Lu and Xu, 2006; Guo et al., 2020; Canovas and Nebreda, 2021). Hyperactivation of these pathways also leads to respiratory disease including asthma, COPD, lung injury, and lung cancer (Arcaroli et al., 2001; Vitos-Faleato et al., 2020). Given their role in mediating homeostasis in the respiratory tract, we sought to determine whether these signaling pathways would be alternatively regulated in response to liquid application (Figure 5A). We observed a significant 122.9-fold ( ± 64.56) increase in p38 MAPK phosphorylation that was accompanied by a 16.5-fold ( ± 3.75) decrease in total p38 protein levels at 24 h of liquid application exposure (Figures 5C, D). Phosphorylation levels of ERK1 were significantly increased after 24 h of liquid application exposure, whereas ERK2 phosphorylation levels were unchanged (Figures 5E, F).

### Liquid application increases pro-inflammatory cytokine and growth factor secretion in dpHBEC cultures

**FIGURE 6:**
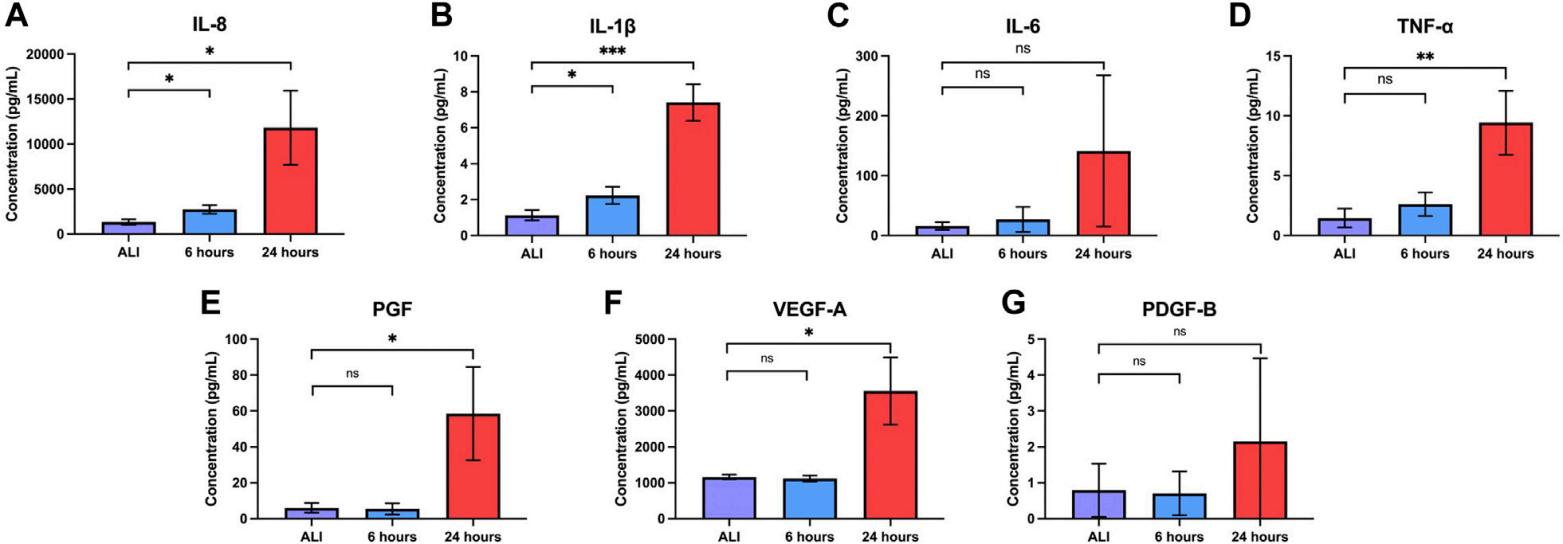
Liquid application increases the release of pro-inflammatory cytokines and growth factors. Effects of 6- and 24-h liquid application on the amount of **(A)** IL-8, **(B)** IL-1b, **(C)** IL-6, **(D)** TNF-a, **(E)** PGF, **(F)** VEGF-A, and **(G)** PDGF-B detected in the basolateral medium. Data represent the mean of *n* = 3 donors and error bars indicate SD. *, **, ***, and ns indicate *p* ≤ 0.05, *p* ≤ 0.01, *p* ≤ 0.001, and not significant, respectively.

Bronchial epithelial cells play a key role in orchestrating inflammation within the respiratory tract in response to inhaled toxicants and other cellular stressors through the secretion of pro-inflammatory cytokines (Hewitt and Lloyd, 2021). After observing the upregulation of pro-inflammatory cytokine transcripts at both 6 and 24 h of liquid application, we sought to determine whether these changes also resulted in the increased release of the corresponding pro-inflammatory cytokine proteins. We observed significant increases in the secretion of IL-8, IL-1β, and TNFα into the basolateral medium at both 6 and 24 h of liquid application exposure (Figures 6A, B, D). While IL-6 secretion exhibited an upward trend after 24 h of liquid application exposure, the change was not statistically significant (Figure 6C). Cytokine concentration and fold change values are reported in Table 1.

**TABLE 1:** Summary of pro-inflammatory cytokine and growth factor ELISA data.

Analyte	ALI concentration (pg/mL)	Concentration at 6 h LAE (pg/mL)	Fold change at 6 h (LAE/ALI)	*p*-value	Concentration at 24 h LAE (pg/mL)	Fold change at 24 h (LAE/ALI)	*p*-value
IL-8	1,338 (±319.9)	2,739 (±482.0)	2.0	0.014	11,820 (±4,116)	8.8	0.012
IL-1β	1.127 (±0.3)	2.233 (±0.5)	2.0	0.027	7.41 (±1.0)	6.6	0.001
IL-6	15.9 (±6.4)	26.88 (±20.9)	1.7	0.434	141.4 (±126.3)	8.9	0.161
TNF-α	1.46 (±0.8)	2.617 (±1.0)	1.8	0.189	9.427 (±2.7)	6.5	0.008
PDGF-B	0.7923 (±0.7)	0.7048 (±0.6)	0.9	0.883	2.157 (±2.3)	2.7	0.385
PGF	6.007 (±2.7)	5.417 (±3.1)	0.9	0.817	58.56 (±26.0)	9.7	0.025
VEGFA	1161 (±64.7)	1119 (±85.9)	1.0	0.532	3,557 (±937.0)	3.1	0.012

Values presented are the mean ( ± SD) of *n* = 3 donors. LAE refers to liquid application exposure.

The regulation of growth factor signaling plays an important role in normal airway homeostasis. Aberrant growth factor signaling occurs in response to chemical exposures and plays a role in airway disease (Desai and Cardoso, 2001; Woo et al., 2004; Voelkel et al., 2006; Wu et al., 2016). Liquid application resulted in the upregulation of several growth factor transcripts at 6 and 24 h so we sought to determine whether changes translated to increased growth factor secretion. We observed significant increases in the secretion of both PGF and VEGF-A, but not PDGF-B, into the basolateral medium after 24 h of liquid exposure (Figures 6E–G). Growth factor concentrations and fold change values are reported in Table 1.

### Liquid application decreases dpHBEC culture epithelial barrier function but not ciliary function

**FIGURE 7:**
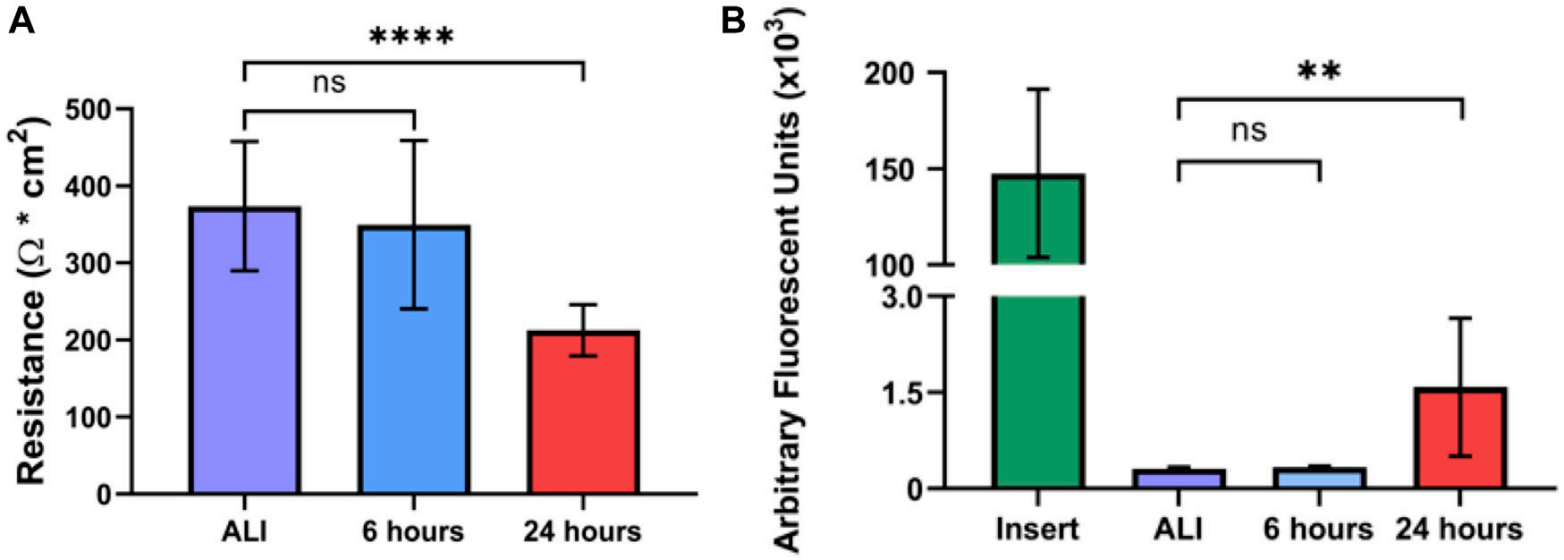
Liquid application reduces epithelial barrier integrity of dpHBEC cultures. **(A)** Effects of 6- and 24-h liquid application on TEER of dpHBEC layer. **(B)** Effects of 6- and 24-h liquid application on permeability of the dpHBEC layer to the translocation of 20 kDa FITC-dextran. Data represent the mean of *n* = 3 donors and error bars represent SD. ****, **, and ns indicate *p* ≤ 0.0001, *p* ≤ 0.01, and not significant, respectively. Data shown represent *n* = 3 donors.

*In vivo*, the normal bronchial epithelium functions in host defense as a physical barrier and a mucociliary escalator. In its role as a barrier, the normal bronchial epithelium forms an electrically resistant tissue layer that is selectively permeable to small molecules that separates inhaled substances from underlying host tissue (Rezaee and Georas, 2014). While often affected by exposure to inhaled insults, loss of bronchial epithelial barrier integrity is also a hallmark of both acute and chronic respiratory disease (Peterson et al., 1993; Gerloff et al., 2017; Smyth et al., 2020; Carlier et al., 2021). Further, loss of epithelial barrier integrity also results in the loss of apical-basolateral polarization and is a key aspect of epithelial-to-mesenchymal transition (EMT), an early step in epithelial carcinogenesis (Saitoh, 2018). We evaluated the effect of liquid application on epithelial barrier integrity by determining the electrical resistance of the epithelial layer by trans-epithelial electrical resistance (TEER). Cultures maintained at ALI exhibited a mean TEER of 373.7 ( ± 83.9) Ω × cm^2^, which was significantly reduced to 212.7 ( ± 33.3) Ω × cm^2^ after 24 h of liquid application (Figure 7A); however, TEER was not significantly affected after 6 h of liquid application (349.7 ± 109.2 Ω × cm^2^). We then evaluated whether the decreased electrical resistance coincided with an increase in the small molecule permeability of the epithelial barrier. In concordance with the observation of decreased TEER, liquid application significantly increased the permeability of the epithelial layer to 20 kDa FITC-conjugated dextran, which is a similar molecular weight to small biomolecules (*e.g.*, IL-6), after 24 h (1581 ± 1076 arbitrary fluorescent units (AFU)), but not at 6 h (329.9 ± 19.6 AFU), when compared to cultures maintained at ALI (310.0 ± 21.9 AFU) (Figure 7B).

The normal *in vivo* bronchial epithelium contains actively beating cilia, a biological feature that is recapitulated *in vitro* by dpHBEC cultures. Despite observing the effects on barrier integrity described above, we did not observe any significant effect of liquid application on CBF or the percentage of active ciliated surface area of dpHBEC cultures (Supplementary Figure S3).

## Discussion

The direct application of experimental treatments in aqueous solvents to differentiated primary human airway epithelial cell cultures is, and has been, a common practice for the dosing of *in vitro* ALI systems with biological stimuli, experimental interventions, and other test substances. Liquid application dosing is considered to be practical due to its expediency, low cost, and minimal technical requirements. The application of liquid to the apical surface of differentiated primary human airway epithelial cell cultures disrupts ALI conditions that are a critical component of their ability to represent the luminal surface of the respiratory tract *in vivo*; however, the effects of different liquid application conditions (*e.g.*, liquid volume, type of solvent, and treatment duration) are poorly understood. The success of initiatives to leverage *in vitro* test systems in respiratory biology, pharmaceutical development, disease research, and inhaled chemical testing depends on building confidence in their ability to represent *in vivo* human-relevant physiology. Additionally, characterizing the effects of liquid application as a dosing modality for ALI-dpHBEC cultures is critical to create context for the interpretation and integration of data collected with this method into applicable decision-making processes.

### Considerations for liquid application conditions

The conditions in which liquid application dosing is used, including the aqueous solvent used, volume of liquid applied, and duration of treatment, vary widely. We selected ALI medium as the applied liquid instead of PBS, 0.9% saline, or water to avoid generating an ionic gradient across the epithelial barrier and reducing nutrient availability. Additionally, while 0.9% saline is commonly used as a vehicle, it does not contain a pH buffer and has a broad pH range (manufacturer specification range of 4.5–7.0), which is often below pH 6.0 based on our evaluation of certificates of analysis from the commonly used manufacturers Baxter (#2F7123/#2F7124, lots #G154987, G139168, G160308, and G140409; mean pH 5.63 at room temperature according to certificates of analysis) and Cytiva (#Z1376, lots #WH30698322, WH30698323, WH30698324, and WH30698325; mean pH 5.79 at room temperature according to certificates of analysis). We used 250 µL of ALI medium in 12 mm Corning Transwell^®^ inserts (221 μL/cm^2^; equal to 73 µL in a 6.5 mm Transwell^®^ insert) for this study because it was the smallest volume that we were able to use while maintaining complete coverage of the apical surface of the dpHBEC culture for the 24-h treatment duration. While smaller volumes are often used in studies involving liquid application dosing, the reduction in apical volume that occurs over the course of the treatment duration results in variable and ultimately discontinuous coverage of the treated culture. The ability of volumes often used to dose ALI cultures, such as 10–50 µL on a 6.5 mm cell culture insert (equivalent to 34–170 µL in the 12 mm inserts used in this study), to achieve and maintain complete coverage of the treated culture over the course has not been characterized. Based on observations reported here (Figure 1B), the use of applied liquid volumes below that used in this study would result in a gradient of liquid coverage that would be expected to lead to inconsistent test substance concentration and flux, ion and/or nutrient concentrations, and exposed oxygen concentrations across the exposed culture. Unfortunately, the potential impact of applied liquid volume on any, or all, of these aspects of experimental exposures remains uncharacterized. Thus, the data collected from these smaller volumes may reflect the impact of either, or both, of these scenarios instead of just the effects of the test substance.

### The application of liquid causes dpHBEC co-cultures to deviate from a normal phenotype

The use of differentiated primary airway epithelial cell systems as an *in vitro* model with enhanced physiological relevance compared to either their undifferentiated counterparts or analogous cell lines is predicated on the assumption that the cultures are comparable to their donor tissue of origin. That is, cultures generated from “normal healthy” donors are representative of the respective region of the respiratory tract *in vivo* in the “normal healthy” population. Here we report that the application of liquid to dpHBEC caused significant transcriptional reprogramming of 4,169 and 10,268 genes, which represented 17.6% and 43.4% of expressed genes, at 6 and 24 h of liquid application, respectively (Figure 2). Pathway analysis of changes in transcriptional profiles, as well as phenotypic and functional endpoints, indicated that the application of liquid to dpHBEC cultures caused effects that were consistent with cancer/epithelial-to-mesenchymal transition (EMT), fibrosis, hypoxia, wound healing, and pro-inflammation (Figures 3, 4). Thus, dpHBEC cultures were no longer consistent with their “normal healthy” counterparts (*i.e.*, incubator controls maintained at ALI) following the application of liquid to their apical surface. In addition to this deviation, liquid application also caused effects similar to those elicited by a broad range of inhaled toxicants within the respiratory tract.

While this study focused on the effects of liquid application on the dpHBEC cell layer, we also observed significant transcriptional reprogramming in IMR90 fibroblasts residing beneath liquid-treated dpHBECs (Supplementary Figures S2-4). While there were only modest transcriptional changes in the fibroblasts by 6 h, more substantial changes occurred by 24 h. At this later time point, the majority of alternatively regulated pathways indicated the development of a pro-carcinogenesis phenotype that complemented observations in the adjacent dpHBECs. Future studies examining the auto- and paracrine signaling events involved in the interactions of these 2 cell types, as well as the intracellular signaling in fibroblasts, will be a valuable advancement to the field.

### Cell signaling

The response of the bronchial epithelium to inhaled toxicants and biological stimuli is mediated through the modulation of cellular signaling pathways, which regulate gene expression, cell survival and growth, and functional properties of the cell layer. Here, we observed that the application of liquid induces a significant increase in HIF-1α protein stabilization at 6 h that is followed by increased p38 and ERK1 phosphorylation at 24 h (Figure 5). Activity of the HIF-1 signaling pathway is minimal under normoxic conditions in the healthy airway epithelium (Shimoda and Semenza, 2011). Under reduced oxygen concentrations, hypoxia-responsive gene expression is upregulated by the activation of the HIF-1 transcription factor following stabilization and nuclear translocation of its regulatory HIF-1α subunit (Dengler et al., 2014). Expression of HIF-1-responsive genes, including VEGF-A, ADM, and GLUT3, play key roles in angiogenesis, metabolism, and cell survival (Masoud and Li, 2015). While the induction of HIF-1 signaling is often considered in the context of tumorigenesis/carcinogenesis, aberrant HIF-1α signaling also occurs in asthma, COPD, lung inflammation, acute lung injury, and ischemic lung injury (Ziello et al., 2007; Shimoda and Semenza, 2011; Ahmad et al., 2012; Masoud and Li, 2015; Tam et al., 2020). HIF-1 activation also results from exposure to inhaled substances including cigarette smoke, particulate matter, and many natural product-derived small molecules (Nagle and Zhou, 2006; Brant and Fabisiak, 2013; Daijo et al., 2016). HIF-1α activation also contributes to EMT and enhanced pro-survival signaling (Chen and Sang, 2016).

Differences in oxygen content affect the physiology of cell culture systems, with a notable role for the activation of HIF-1-dependent signaling (Shimoda and Semenza, 2011; Masoud and Li, 2015). The availability of oxygen depends upon factors such as medium volume, culture configuration, cell type, and seeding density (Stuart et al., 2018). While medium volume affects diffusion of ambient oxygen to the cells, cell type and seeding density both influence consumption of available dissolved oxygen (Stuart et al., 2018). dpHBEC cultures grown under ALI cultures experience an oxygen concentration that is similar to the lumen of the respiratory tract *in vivo*, which is critical to their differentiation under ALI conditions (Kouthouridis et al., 2021). The increase in oxygen exposure that follows the transition of pHBEC cultures to ALI conditions is also accompanied by decreased HIF-1α protein levels (Klasvogt et al., 2017; Kouthouridis et al., 2021). The importance of oxygen concentration on pHBEC differentiation has been further indicated by the demonstration that pHBECs grown in a submerged system with greater oxygen availability results in a phenotype identical to dpHBECs grown at ALI (Kouthouridis et al., 2021). In contrast, here we have demonstrated that the reduction of available oxygen by the application of liquid to dpHBEC-ALI cultures appears to initiate their dedifferentiation, which coincides with the stabilization of HIF-1α. Future studies are required to characterize the relationship between oxygen availability, HIF-1 activation, dpHBEC culture physiology and exposure outcomes in the context of liquid application exposures.

ERK and p38 have diverse biological functions that include responding to cellular stress as well as promoting cell growth and survival (Roux and Blenis, 2004; Zarubin and Han, 2005; Lu and Xu, 2006; Martínez-Limón et al., 2020). The alternative regulation of cellular signaling proteins such as ERK1 and p38, alone or in concert, has been observed in response to exposures to a broad range of inhaled toxicants including ozone, diesel exhaust particulates, acrolein, asbestos, cigarette smoke, and diacetyl (2,3-butanedione) (Hashimoto et al., 2000; Cummins et al., 2003; Moretto et al., 2012; McCullough et al., 2014; Xu et al., 2015; Kelly et al., 2019). Further, the alternative regulation of these signaling proteins also occurs in a variety of respiratory diseases including COPD, asthma, fibrosis, EMT, and cancer (Yoshida et al., 2002; Renda et al., 2008; Alam and Gorska, 2010; Gui et al., 2012; Madala et al., 2012). Similar to HIF-1, the activation of these signaling proteins by liquid application alone has the potential to confound exposure outcomes and interpretation. For example, the activation of ERK and p38 by the application of liquid alone may have an additive or synergistic effect on the activation of these proteins, or other signaling pathways, that occurs as a result of exposure to a test substance. Thus, exposure to the test substance by liquid application could enhance toxicity-related exposure outcomes that are dependent on ERK and/or p38 activation such as the induction of pro-inflammatory gene expression in pHBEC cultures (McCullough et al., 2014; Bowers et al., 2018). Alternatively, the role of HIF-1α, ERK, and/or p38 activation in pro-survival and growth signaling may mean that the activation of these pathways by the application of liquid alone could diminish the cytotoxic effects of a test substance applied by liquid dosing. The pleiotropic effects of HIF-1α, ERK, and p38 provide many possible avenues by which the effect of liquid application could impact the outcome and/or interpretation of chemical testing studies that utilize liquid dosing of dpHBEC-ALI cultures.

### Induction of pro-inflammatory mediators

The airway epithelium plays a central role in the release of pro-inflammatory cytokines that play concerted roles in the response to inflammatory stimuli, airway insult/injury, and tissue homeostasis. While beneficial in the resolution of damage, their dysregulation results in airway remodeling, a chronic inflammatory state, impaired lung function, and disease (Reynolds et al., 2018). Given the role of inflammation and response to tissue injury in the etiology of toxicant-induced effects, several pro-inflammatory cytokines, such as IL-8 and TNF-α, are common endpoints in the evaluation of chemical toxicity. In the study reported here, the application of liquid alone induced significant increases in the release of IL-8 and IL-1β at 6 h and IL-8, IL-1β, and TNF-α at 24 h (Figure 6). The increased release of these pro-inflammatory cytokines has been implicated in a variety of respiratory diseases including COPD, acute lung injury, epithelial-to-mesenchymal transition, and squamous cell metaplasia (Chung, 2001; Goodman et al., 2003; Herfs et al., 2012; Pain et al., 2014). Further, the dysregulation of pro-inflammatory cytokine secretion also results from exposure to a diverse range of inhaled substances including diesel exhaust particulates, ambient particulate matter, acrolein, cigarette smoke, e-cigarette flavorings, and zinc oxide nanoparticles (Stoehr et al., 2015; Zhou et al., 2015; Gerloff et al., 2017; Murray et al., 2017; Grytting et al., 2022). Therefore, the observation that liquid application alone significantly enhances pro-inflammatory cytokine secretion indicates that the effects of this dosing method alone can induce a disease-like phenotype and could limit the identification of test substances that induce a pro-inflammatory response in the bronchial epithelium. Further, the induction of pro-inflammatory mediators by liquid application alone could account for the significantly lower levels of IL-8 induction in dpHBEC exposed to reactive aldehydes by liquid application dosing compared to matched ALI dosing reported by Dwivedi *et al* (Dwivedi et al., 2018). The increased secretion of pro-inflammatory mediators by the bronchial epithelium also has the potential to prime the culture to respond more strongly to test substance exposure compared to the epithelium at ALI or the *in vivo* airway. For example, HTB-54 lung epithelial cells primed with TNF-α, were found to secrete more IL-8 following particulate matter exposure compared to non-primed cells (Ning et al., 2004). Additionally, the detection of elevated IL-8, TNF-α, or IL-1β is considered a biomarker for a wide variety of inflammatory conditions *in vivo* (Shahzad et al., 2010; Andreasson et al., 2017; Aghapour et al., 2018; Tutkun et al., 2019). Thus, the use of liquid application dosing may invalidate the use of pro-inflammatory cytokine release as an *in vitro* biomarker that is directly linked to *in vivo* mediators of toxicity.

### Airway epithelial barrier integrity

The healthy bronchial epithelium functions as the barrier that protects the underlying lung tissue from inhaled pathogens, chemicals, and other materials. We observed that the application of liquid to dpHBEC significantly decreased barrier integrity and increased permeability as measured by a reduction in TEER and an increase in 20 kDa FITC-Dextran permeability at 24 h, respectively (Figure 7). Loss of epithelial barrier integrity is a hallmark of airway injury and occurs in airway diseases including COPD, acute respiratory distress syndrome, asthma, fibrosis, and EMT/cancer, thus supporting the conclusion that the application of liquid to dpHBEC causes a phenotype that is consistent with a range of respiratory diseases (Georas and Rezaee, 2014; Lamouille et al., 2014; Brune et al., 2015; Aghapour et al., 2018; Carlier et al., 2021). In contrast, hypoxia is protective against oxidant-induced loss of barrier integrity (Olson et al., 2011); however, our observations indicate that the stabilization of HIF-1α protein alone (Figure 5) is not sufficient to prevent the loss of barrier integrity following liquid application. Epithelial barrier integrity is also adversely affected following exposure to a diverse range of inhaled substances including diesel exhaust particulates, ambient particulate matter, acrolein, asbestos, ozone, wood smoke particulates, and e-cigarette flavorings (Peterson et al., 1993; Yu et al., 1994; Olson et al., 2011; Gerloff et al., 2017; Xiong et al., 2018; Zeglinski et al., 2019; Smyth et al., 2020; Xian et al., 2020; Bowers et al., 2022). Thus, the observation that liquid application alone significantly reduces barrier integrity indicates the potential for this manner of dosing to limit the sensitivity of this approach to identify test substances that disrupt barrier function.

The polarized nature of the respiratory epithelial barrier results in the segregation of receptors and other factors between their apical and basolateral surfaces, which is important in maintaining tissue homeostasis. Liquid-induced disruption of barrier integrity could result in aberrant distribution of both cell surface receptors and/or the test substance relative to exposures conducted under ALI conditions or by inhalation *in vivo*. For example, members of the human epidermal growth factor receptor (HER) family are differentially localized to the apical and basolateral compartments of the airway epithelium, which plays a critical role in their function *in vivo* (Brune et al., 2015). Under homeostatic conditions, the epidermal growth factor receptor (EGFR; dimer of HER1) and the heterodimeric HER2/3 receptor are localized to the basolateral surface of the airway epithelium where they are separated from their ligands, epidermal growth factor (EGF) and heregulin, respectively, which are expressed in the apical compartment (Takeyama et al., 2001; Vermeer et al., 2003). When this compartmentalization is disrupted by damage to the epithelium, EGF and heregulin are able to access their respective receptors to promote repair of the epithelium through cell proliferation, differentiation, and protection (van der Geer et al., 1994; Walker, 1998; Yarden and Sliwkowski, 2001; Brune et al., 2015). Further, members of the toll-like receptor (TLR) family are also selectively localized to the apical or basolateral surfaces of airway epithelial cells where they play distinct roles in the recognition of exogenous pathogen-associated molecular patterns (PAMPs) and endogenous damage-associated molecular patterns (DAMPs) (Ioannidis et al., 2013; Arora et al., 2019). Activation of the HER and TLR families are involved in various diseases of the respiratory tract and are activated in response to a wide range of inhaled chemicals/materials as well as DAMPs resulting from the effects of reactive oxygen species that are common mediators of inhaled toxicants (Lucas and Maes, 2013; Luettich et al., 2017; Arora et al., 2019). Thus, loss of barrier integrity resulting from liquid application could result in the artificial exposure of test substances to receptors that they would not interact with under ALI conditions. Liquid application-induced decompartmentalization could also induce a pro-repair response that could limit the effects of test substances on cell survival. Further, activation of EGFR due to loss of epithelial barrier function also promotes mucus production (Burgel and Nadel, 2004), which could be interpreted as an adverse effect of exposure and/or alter the interaction of the test substance with the epithelial cell surface. Importantly, these effects may not be easily accounted for by the normalization of the effects of test substance exposures to vehicle controls.

### Potential impact of a hypertonic environment

The reduction of apical volume occurring during the treatment duration observed in Figure 1B may result from evaporation, para- or transcellular transport, or both. If occurring by evaporation, this change in apical volume will result in a hypertonic environment. We were unable to find any reports describing the effect of apical solvent evaporation in air-liquid interface *in vitro* respiratory tract cultures; however, the effects of exposure to hypertonic saline *in vitro* and *in vivo* have been previously reported. Nebulized hypertonic saline (HS) increases mucus clearance *in vivo* by increasing airway hydration and reduced viscosity through the disruption of ionic bonds and induced conformational changes in mucus proteins (Ziment, 1978; Robinson et al., 1997; Graeber et al., 2013). Treatment-induced increases in airway hydration were supported by the observation that aerosolized HS increased airway surface liquid (ASL) height in dpHBEC-ALI cultures *in vitro* (Goralski et al., 2018). Unfortunately, we did not evaluate mucus viscosity or ASL height in this study; however, understanding the impacts of liquid application and solvent evaporation on these parameters would be valuable given the potential for mucus to trap and sequester inhaled toxicants. Changes in ASL volume may also impact test substance concentrations, especially when experimental treatments are delivered in low applied liquid volumes. While hypertonic saline did not affect inflammation in a mouse model of chronic obstructive lung disease (Graeber et al., 2013), it did attenuate inflammation following trauma and hemorrhagic shock in rats (Wohlauer, et al., 2012) and pro-inflammatory gene expression following treatment of primary human small airway epithelial cells with TNF-α, IL-1β, and interferon (IFN)-γ *in vitro* (Mitra et al., 2017). While we expected some level of evaporation to occur during these treatments, we observed that the effects of liquid application increased the expression and secretion of pro-inflammatory cytokines (Figures 3, 4, 6). Thus, our observations indicate that either the evaporation in our approach did not create a sufficiently hypertonic condition, or the anti-inflammatory potential of hypertonic conditions is not sufficient to prevent the induction of pro-inflammatory mediators the results from liquid application. Additional studies are required to determine whether the mitigation of pro-inflammatory stimuli by hypertonic conditions described by Mitra et al. (2017) alters the ability of low-volume liquid application dosing approaches to represent the induction of pro-inflammatory mediators by test substances.

### Study limitations

The study reported here provides novel insight into the effects of liquid application on dpHBEC but has limitations that should be considered. First, we have previously shown that some endpoints vary based on inter-individual variability (Bowers et al., 2018; 2022). While the range of inter-individual variability for the endpoints evaluated here has not been evaluated, this study utilized an *n* of three dpHBEC donors for each endpoint evaluated. It remains unclear whether this sample size is sufficient to fully reflect the range of variability in the effects of liquid application on dpHBEC in the “normal healthy” donor population. Further, future studies using donor-matched primary HLF will be required to address the potential for inter-individual variability on the effects of liquid application on HLF beneath the epithelial barrier. Second, the liquid application dosing method has been used with a broad range of applied liquid volumes and in different permeable cell culture insert configurations (*e.g.*, manufacturers, insert diameters, pore sizes, pore densities, membrane materials, *et cetera*). The observations reported here only evaluated one set of conditions. Third, different aqueous solvents including water, 0.9% saline, buffered (phosphate or other) saline, or cell culture medium have been used in reported studies that utilized liquid application dosing.

The impact of the type, volume, and duration of liquid application on the effects observed in dpHBEC cultures should be evaluated in future studies to provide a more comprehensive understanding of these variables on *in vivo* physiology-relevant endpoints, exposure outcomes, and test substance delivery/dosimetry. While this study sought to describe the effects of liquid application on the dpHBEC-ALI system, future studies could also provide a thorough characterization of the molecular mechanisms underlying the effects reported here. More specifically, additional work could more comprehensively evaluate the hypotheses generated with the RNA-sequencing data regarding the alternative regulation of biological pathways by liquid application. Additional studies could also determine the relative contributions of the MAPK pathways and HIF1α signaling in the response of dpHBEC-ALI cultures to liquid application. Further studies are also required to determine whether differentiated primary human airway epithelial cell ALI models of other regions of the respiratory tract are similarly affected by the application of liquid alone.

## Conclusion

**FIGURE 8:**
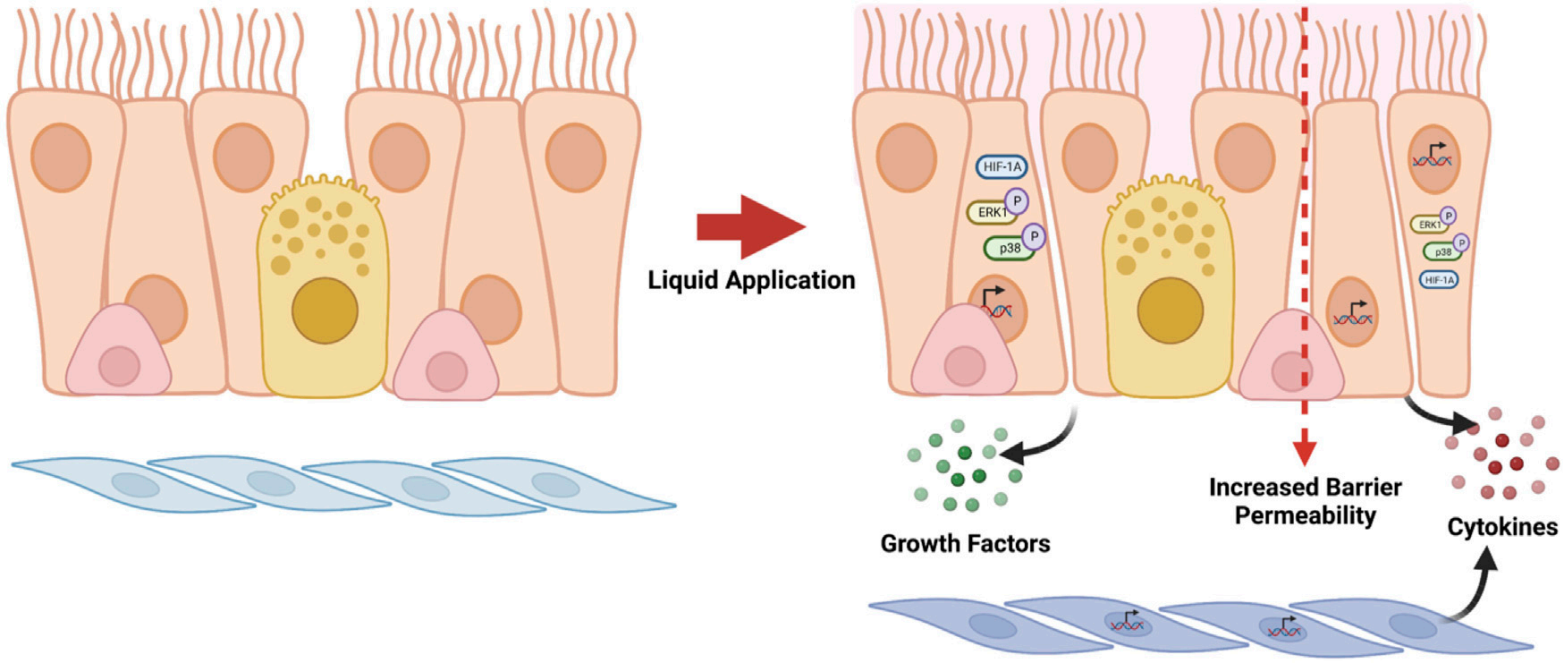
Summary of effects caused by liquid application to dpHBEC-ALI cultures. The application of 250 µL ALI medium induces the alternative regulation of transcripts, the activation of stress-responsive protein signaling pathways, the secretion of pro-inflammatory cytokines and growth factors, and the breakdown of epithelial barrier integrity. Figure made with BioRender.com.

*In vitro* studies that utilize differentiated primary human airway cell systems to evaluate inhalable test substances frequently rely on direct application dosing; however, publications describing these studies rarely report the use of an ALI control (*i.e.*, not subjected to liquid application) to account for the effects of the application of liquid alone in the data analysis and interpretation. The findings described here demonstrate that the application of liquid alone can cause large-scale reprogramming of gene expression in differentiated ALI cultures and significant changes to aspects of the cell culture physiology that are of great importance with respect to the ability of the dpHBEC-ALI system to represent key aspects of *in vivo* respiratory tract physiology. Importantly, the effects liquid application on culture physiology occurred in the absence of changes in the cultures that would be detectable by basic visual microscopic examination. Thus, they would likely go unnoticed without more thorough evaluation. Further, the changes that result from liquid application involve the alternative regulation of cellular pathways that are often involved in mediating the response to exogenous stimuli and diseases of the respiratory tract. The endpoints that we observed to be alternatively regulated by liquid application are also commonly used to identify and quantify the toxicity or efficacy of test substances (*i.e.*, epithelial barrier function, cellular signaling pathway activation, pro-inflammatory cytokine release, and growth factor production) (Figure 8).

The findings reported here support the conclusion that the impact of the application of liquid alone has the potential to confound the accuracy, sensitivity, and translation of data resulting from the use of liquid dosing of dpHBEC-ALI systems for respiratory biology research and the evaluation of inhaled substances. Further, given the observations reported here, the comparison of test substance exposures to their respective vehicle treatment may not be sufficient to normalize data for comparison or extrapolation to *in vivo* human exposure outcomes. Additional studies are required to characterize the impact of liquid application on ALI cultures and exposure outcomes and provide a better understanding of the impact of liquid application on exposure outcomes and comparability of liquid application and ALI dosing outcomes in differentiated primary human airway epithelial tissue models.

The use of differentiated ALI cultures to inform inhaled chemical hazard identification and human health risk assessment is in its relative infancy, but the demand of the use of the approach is increasing. This is evidenced by the use of this dosing method in a recent case study for the use of an ALI-differentiated *in vitro* system as a part of an integrated approach to testing and assessment (IATA) to refine an inhalation risk assessment by the Organisation for Economic Co-operation and Development (OECD) (National Research Council, 2007; United States Environmental Protection Agency, 2020), an organization that develops international guidelines for studies used to evaluate pharmaceuticals, as well as commercial and industrial chemicals. Identification of considerations and limitations to the use of direct application dosing of ALI cultures at this early stage is critical for defining the context for their use in the assessment of inhalable substances. It also provides guidance on important aspects of study design and reporting, as well as considerations for the interpretation of data derived from liquid application dosing of dpHBEC-ALI systems, and potentially differentiated primary cell-based ALI models of other regions of the human respiratory tract. Additional studies are needed to determine whether the effect of liquid application alone impacts the outcomes of *in vitro* inhaled chemical testing, whether the effects observed here are specific to the conditions used in this study, and if these effects apply to differentiated ALI models of other regions of the human respiratory tract. Future studies are also needed to determine whether aspects of this dosing method (*e.g.*, type of aqueous buffer used, liquid volume, exposure duration, *et cetera*) can be optimized to minimize the effects on culture physiology. Ultimately, improving our understanding of how key aspects of differentiated ALI models of the human respiratory tract impact their physiology, response to test substance exposures, and data analysis and interpretation is central to building confidence in their ability to reflect *in vivo* human outcomes and advancing the translation of data from these *in vitro* systems into decision making.

## Data Availability

The datasets presented in this study can be found in online repositories. The names of the repository/repositories and accession number(s) can be found below: https://www.ncbi.nlm.nih.gov/geo/, GSE198884. https://catalog.data.gov/dataset/epa-sciencehub, by searching the manuscript title or DOI: 10.23719/1526421.
