# Supplementary material for: Liquid application dosing alters the physiology of air-liquid interface (ALI) primary human bronchial epithelial cell/lung fibroblast co-cultures and in vitro testing relevant endpoints
Source: Front Toxicol. 2024 Jan 23;5:1264331. doi: 10.3389/ftox.2023.1264331 (PMC10922929; doi:10.3389/ftox.2023.1264331)
Supplement: Supplementary file 1 [file Presentation1.pdf]

Supplementary Materials

Liquid Application Dosing Alters the Physiology of Air-Liquid Interface Primary Bronchial Epithelial Cell/Lung Fibroblast Co-Cultures and *In Vitro* Testing Relevant Endpoints

Nicholas M. Mallek[1], Elizabeth M. Martin[2], Lisa A. Dailey[3], and Shaun D. McCullough[3,4*]

1: Curriculum in Toxicology & Environmental Medicine, UNC-Chapel Hill, Chapel Hill, NC, USA
2: Epigenetics & Stem Cell Biology Laboratory, National Institute of Environmental Health Sciences, National Institutes of Health, Department of Health and Human Services, Durham, NC, USA
3: Public Health & Integrated Toxicology Division, Center for Public Health & Environmental Assessment, United States Environmental Protection Agency, Chapel Hill, NC, USA
4: Exposure and Protection, RTI International, Durham, NC, USA
*: corresponding author

Corresponding Author:
Shaun D. McCullough, PhD
3040 E. Cornwallis Road
P.O. Box #12194
Durham, NC 27709
smccullough@rti.org
1-919-248-4595 (office)
ORCID ID: 0000-0001-6660-346X

Competing Financial Interests: The authors declare they have no actual or potential competing financial interests.

Keywords: in vitro to in vivo extrapolation (IVIVE), new approach methods (NAMs), inhalation risk assessment, air-liquid interface (ALI)

Running head: Liquid application alters ALI cultures

Supplementary Table 1

Supplementary Table 1. Sex, self-identified race, and age range of pHBEC donors used in this study.

	Sex	Race	Age Range (yr)
Donor 1	Male	Caucasian	36-40
Donor 2	Male	Black	30-35
Donor 3	Male	Black	30-35
Donor 4	Male	Asian	36-40
Donor 5	Male	Asian	36-40
Donor 6	Male	Caucasian	36-40

Supplementary Table 2

Supplementary Table 2. Primer and probe sequences used for gene expression analysis.

qPCR Target		Sequence
IL-8	Forward	TTGGCAGCCTTCCTGATTTC
	Reverse	TATGCACTGACATCTAAGTTCTTTAGC
	Probe	CCTTGGCAAAACTGCACCTTCACACA
IL-1α	Forward	GACGCCCTCAATCAAAGTATAATTC
	Reverse	CCATGTCAAATTTCACTGCTTCATC
	Probe	TGATCAGTACCTCACGGCTGCTGCA
COX-2	Forward	GAATCATTCACCAGGCAAATTG
	Reverse	TCTGTACTGCGGGTGGAACA
	Probe	TCCTACCACCAGCAACCCTGCCA

Supplementary Table 3

Supplementary Table 3. Antibodies used for immunoblotting.

Antibody	Catalog	Source
phosphoNFkB p65 (Ser536) Rabbit mAb	3033S	Cell Signaling Technologies
NFkB p65 XP Rabbit mAb	8242S	Cell Signaling Technologies
Phospho p44/42 MAPK (ERK1/2) (Thr202/Tyr204) Rabbit mAb	4370S	Cell Signaling Technologies
P44/42 MAPK (ERK1/2) Rabbit mAb	4695S	Cell Signaling Technologies
Phospho-P38 MAPK (Thr180/Tyr182) Rabbit mAb	4511S	Cell Signaling Technologies
P38 MAPK Rabbit mAb	9212S	Cell Signaling Technologies

Supplementary Figure 1

Cell Line Authentication Service
STR Profile Report

ATCC

FTA Barcode: STRB0205

ATCC Sales Order: SO0425743

Test Results for Submitted Sample					ATCC Reference Database Profile			
**Locus**	**Query Profile: IMR90**				**Database Profile: IMR-90; Lung Fibroblast; Human (Homo sapiens)**			
**D3S1358**	[redacted]	[redacted]			[redacted]	[redacted]		
**TH01**	8	9.3			8	9.3		
**D21S11**	[redacted]	[redacted]			[redacted]	[redacted]		
**D18S51**	[redacted]	[redacted]			[redacted]	[redacted]		
**Penta_E**	[redacted]	[redacted]			[redacted]	[redacted]		
**D5S818**	12	13			12	13		
**D13S317**	11	13			11	13		
**D7S820**	9	12			9	12		
**D16S539**	10	13			10	13		
**CSF1PO**	11	13			11	13		
**Penta_D**	[redacted]	[redacted]			[redacted]	[redacted]		
**Amelogenin**	X				X			
**vWA**	16	19			16	19		
**D8S1179**	[redacted]	[redacted]			[redacted]	[redacted]		
**TPOX**	8	9			8	9		
**FGA**	[redacted]	[redacted]			[redacted]	[redacted]		
**D19S433**	[redacted]	[redacted]			[redacted]	[redacted]		
**D2S1338**	[redacted]	[redacted]			[redacted]	[redacted]		
Number of shared alleles between query sample and database profile:								17
Total number of alleles in the database profile:								17
Percent match between the submitted sample and the database profile:								100
The allele match algorithm compares the 8 core loci plus amelogenin only, even though alleles from all loci will be reported when available.								
**NOTE:** *Loci highlighted in grey (8 core STR loci plus Amelogenin) can be made public to verify cell identity. In order to protect the identity of the donor,* ***please do not publish*** *the allele calls from all the STR loci tested. Electropherograms showing raw data are attached.*								

Explanation of Test Results
Cell lines with 80% match are considered to be related; i.e., derived from a common ancestry. Cell lines with between a 55% to 80% match require further profiling for authentication of relatedness.

[ ] The submitted sample profile is human, but not a match for any profile in the ATCC STR database.

[X] The submitted profile is an exact match for the following ATCC human cell line(s) in the ATCC STR database (8 core loci plus Amelogenin): CCL-186

[ ] The submitted profile is similar to the following ATCC human cell line(s):

[ ] An STR profile could not be generated.

Additional Comments:

Submitted sample, STRB0205 (IMR90), is an exact match to ATCC cell line CCL-186 (IMR-90).

Supplementary Figure 1. STR authentication of the IMR90 cell line. As per guidance indicated in the "NOTE" section of the report, calls from alleles indicated in white have been redacted.

Supplementary Figure 2

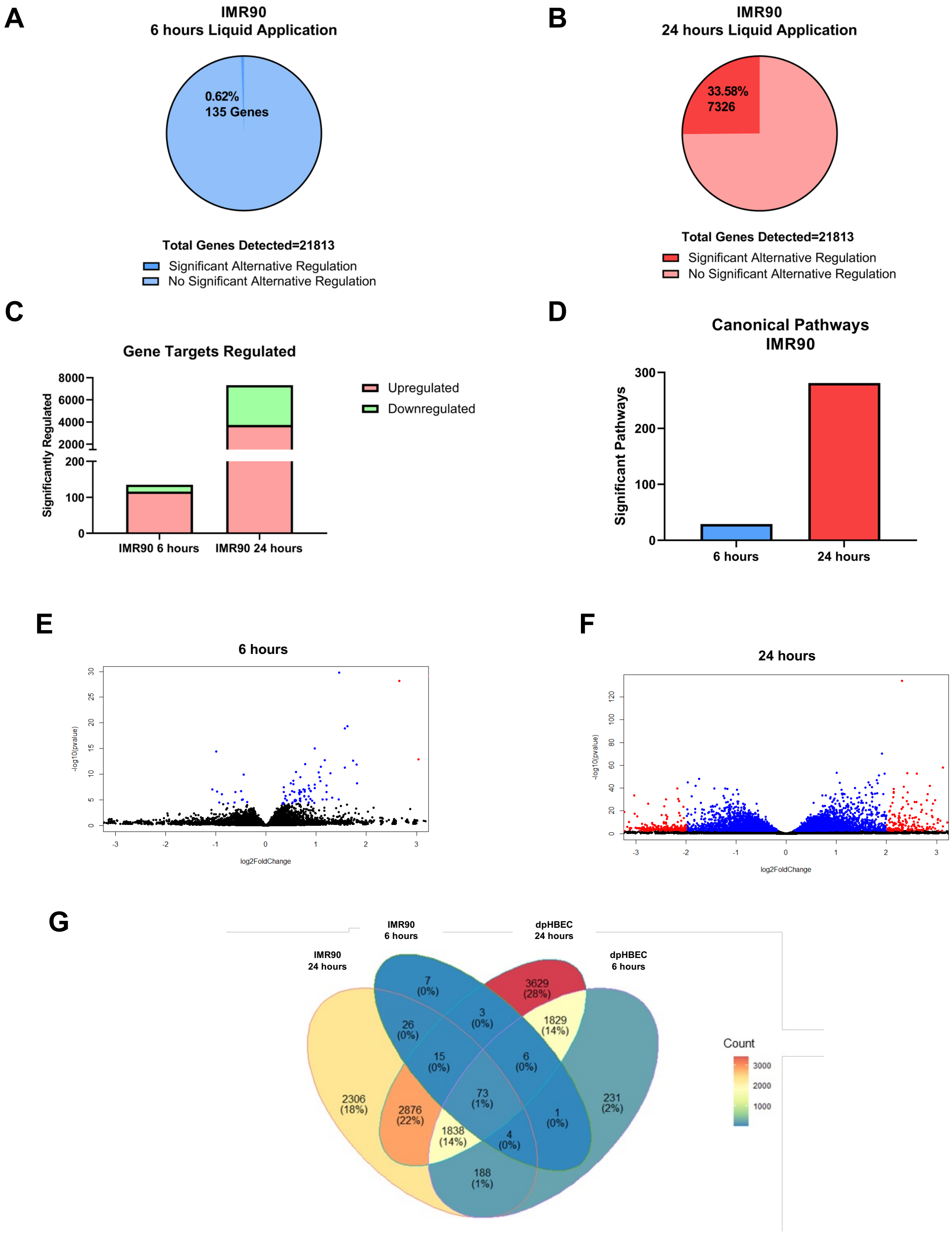

Supplementary Figure 2. Global transcriptome changes following liquid application exposure for 6 or 24 hours. (A-B) The total amount of significantly alternatively regulated genes (adjusted *p*-value <0.05) after 6 hours (A) or 24 hours (B) of liquid application, respectively. (C) The total number of significant alternatively regulated genes from (A-B) separated into upregulated or downregulated after 6 hours or 24 hours of liquid application. (D) Total number of canonical pathways of significant alternatively regulated genes from (A-B) after 6 or 24 hours of liquid application. (E-F) Volcano plots of the gene datasets described in (A-B). Blue points have an adjusted *p*-value of <0.01, and red points have an adjusted *p*-value of <0.05 and a $\log_2$(foldchange) >1. (G) Venn Diagram comparison displaying the number of significant (adjusted *p*-value <0.05) unique and shared genes between the 6- and 24-hour liquid application dataset for both dpHBECs and IMR90s. Data represent *n*=3 donors.

Supplementary Figure 3

A

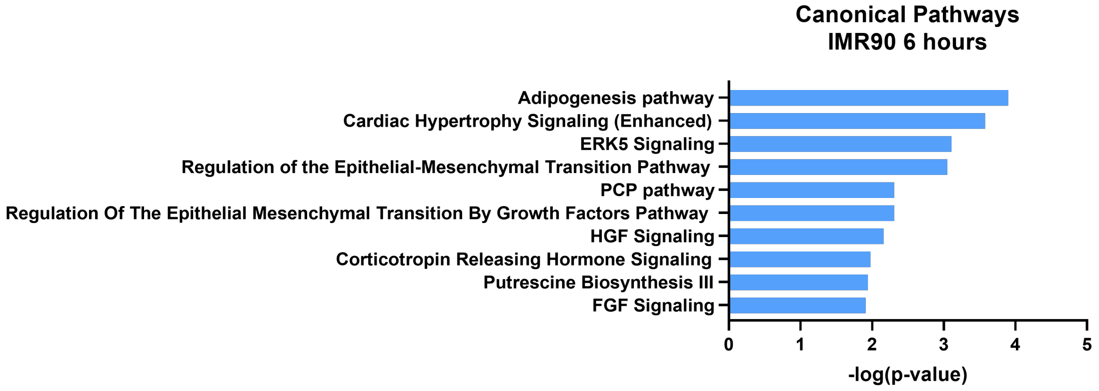

B

Adipogenesis Pathway

Gene	Expression
KLF5	2.19
CEBPD	1.96
HDAC5	1.85
LPIN1	-1.38
NR1D2	-1.53
FZD8	-1.90

Cardiac Hypertrophy

Gene	Expression
PTGS2	3.51
ADRA1D	3.06
MAP3K8	2.81
FGF5	2.10
PDE7B	1.95
HDAC5	1.85
FGF7	1.69
MEF2D	1.46
IGF1R	1.28
WNT7B	-1.55
FZD8	-1.90

ERK5 Signaling

Gene	Expression
MAP3K8	2.81
SGK1	1.84
MEF2D	1.46
GAB1	1.34

Regulation of Epithelial-Mesenchymal Transition

Gene	Expression
FGF5	2.10
FGF7	1.69
ETS1	1.36
GAB1	1.34
WNT7B	-1.55
FZD8	-1.90

PCP Pathway

Gene	Expression
PRICKLE1	1.79
WNT7B	-1.55
FZD8	-1.90

Regulation of EMT by Growth Factors

Gene	Expression
FGF5	2.10
FGF7	1.69
LATS2	1.61
ETS1	1.36
GAB1	1.34

HGF Signaling

Gene	Expression
PTGS2	3.51
MAP3K8	2.81
ETS1	1.36
GAB1	1.34

Corticotropin Releasing Hormone Signaling

Gene	Expression
NR4A1	6.31
PTGS2	3.51
NPR3	2.97
MEF2D	1.46

Putrescine Pathway

Gene	Expression
ODC1	-1.32

FGF Signaling

Gene	Expression
FGF5	2.10
FGF7	1.69
GAB1	1.34

Supplementary Figure 3. Ingenuity pathway analysis of genes exhibiting a significant ± log2 fold change in IMR90 fibroblasts after 6 hours of liquid application on dpHBEC-ALI cultures. (A) The top 10 most significant canonical pathways by the –log(p-value) identified after 6 hours of liquid application. (B) The top genes, by absolute value, in each canonical pathway identified in (A), and their respective fold change expression over ALI cultures. Blue targets are cell signaling-related and yellow are inflammation-related.

Supplementary Figure 4

A

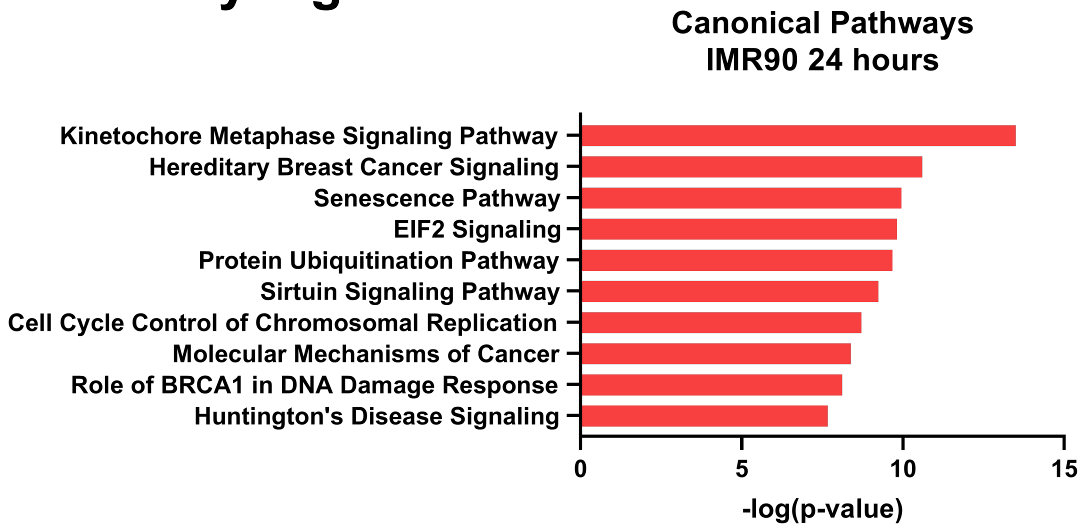

B

Kinetochore Metaphase Signaling	
**Gene**	**Expression**
SKA1	-10.56
NDC80	-8.54
CASC5	-8.04
CDC20	-7.78
BIRC5	-7.58
SPC25	-7.51
SKA3	-7.16
ZWINT	-7.06
NUF2	-6.55
CENPU	-6.11

Hereditary Breast Cancer Signaling	
**Gene**	**Expression**
CDC25C	-6.72
CDK1	-5.00
BLM	-4.94
FANCD2	-4.84
CCNB1	-4.63
BRCA1	-3.81
RFC3	-3.47
H2AFX	-3.19
BRCA2	-2.87
PIK3R3	-2.86

Senescence Pathway	
**Gene**	**Expression**
IL8	14.58
CACNG6	7.03
IL6	6.90
CDC25C	-6.72
E2F8	-5.68
MAPK15	5.30
CDK1	-5.00
IL1A	4.73
CCNB2	-4.73
CCNB1	-4.63

EIF2 Signaling Pathway	
**Gene**	**Expression**
BCL2	-3.50
ATF3	3.03
PIK3R3	-2.86
RPS10	-2.22
NOX4	2.09
RPS27	2.04
ATF5	-2.00
EIF5	-2.00
AKT3	1.95
PIK3C2B	-1.93

Protein Ubiquitination Pathway	
**Gene**	**Expression**
CDC20	-7.78
UBE2C	-4.92
HLA-G	4.40
BRCA1	-3.81
HSPA2	3.12
BIRC3	2.97
HLA-E	2.43
DNAJB4	2.29
HLA-B	2.05
DNAJC28	-2.05

Sirtuin Signaling Pathway	
**Gene**	**Expression**
IL8	14.58
HIST1H1E	-11.49
PPARGC1A	-6.77
MAPK15	5.30
FOXO1	4.41
LDHD	3.72
ATG16L2	2.97
GABARAPL1	2.57
PCK2	-2.51
E2F1	-2.43

Cell Cycle Control of Chromosome	
**Gene**	**Expression**
TOP2A	-5.74
CDK1	-5.00
CDK18	4.68
CDC6	-4.38
CDC45	-4.24
ORC1	-3.57
POLA1	-3.16
MCM4	-3.14
MCM5	-2.81
PRIM1	-2.80

Molecular Mechanisms of Cancer	
**Gene**	**Expression**
ADCY4	6.75
CDC25C	-6.72
BMP2	6.29
E2F8	-5.68
ITGA2B	5.26
CDK1	-5.00
FANCD2	-4.84
CDK18	4.68
FOXO1	4.41
CAMK2B	4.18

Role of BRCA1 in DNA Damage Response	
**Gene**	**Expression**
PLK1	-5.77
E2F8	-5.68
BLM	-4.94
FANCD2	-4.84
E2F2	-4.00
BRCA1	-3.81
RFC3	-3.47
BRCA2	-2.87
RBL1	-2.77
FANCA	-2.71

Huntington's Disease Signaling	
**Gene**	**Expression**
CASP12	5.57
PRKCG	4.65
HSPA2	3.12
PIK3R3	-2.86
CAPN3	-2.83
HDAC5	2.38
HDAC9	2.32
AKT3	1.95
PIK3C2B	-1.93
HSPA1A	-1.88

Supplementary Figure 4. Ingenuity pathway analysis of genes exhibiting a significant ± log2 fold change in IMR90 fibroblasts after 24 hours of liquid application on dpHBEC-ALI cultures. (A) The top 10 most significant canonical pathways by the –log(p-value) identified after 24 hours of liquid application. (B) The top 10 genes, by absolute value, in each canonical pathway identified in (A), and their respective fold change expression over ALI cultures. Blue targets are cell signaling-related and yellow are inflammation-related.

Supplementary Figure 5

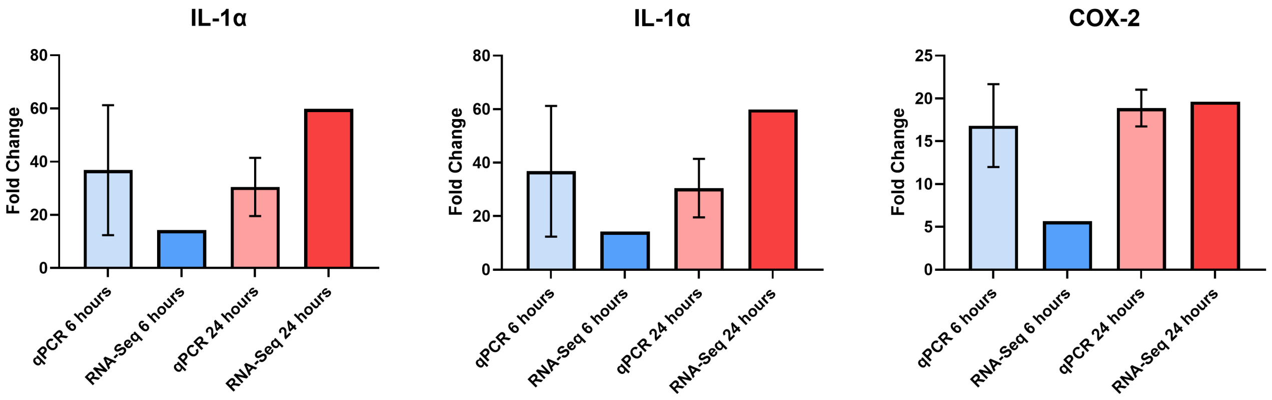

Supplementary Figure 5. qPCR validation of three pro-inflammatory transcripts identified as highly upregulated by RNA-Sequencing following liquid application for either 6 or 24 hours. qPCR data are presented as the mean of *n*=3 donors, and error bars represent SD.

Supplementary Figure 6

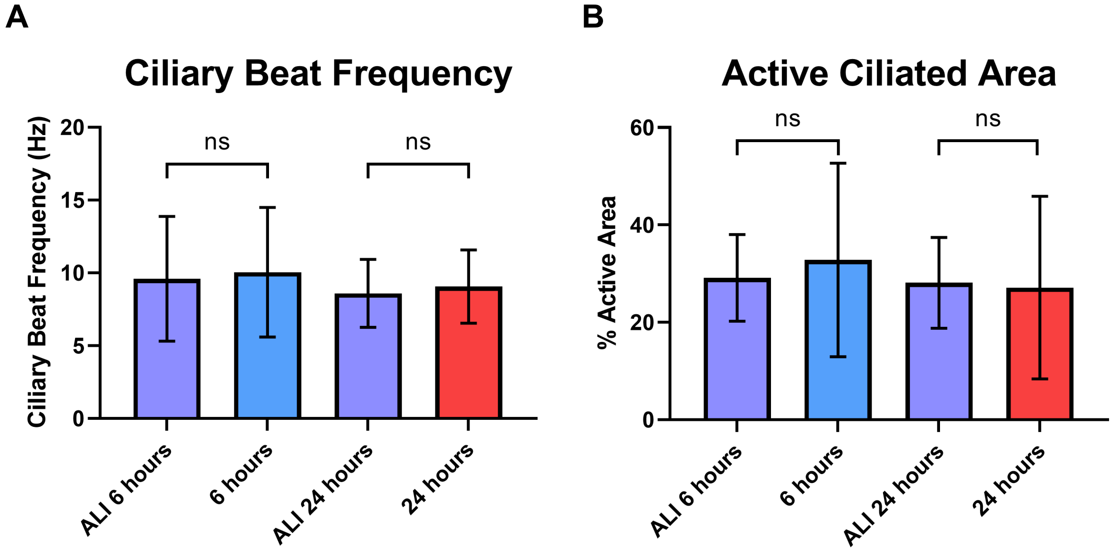

Supplementary Figure 6. Ciliary beat frequency (CBF) and active ciliated area of pHBEC-ALI cultures following either 6 or 24 hours of liquid application. (A) There was no change in CBF after either 6 or 24 hours of liquid application. (B) There was no change in active ciliated area after either 6 or 24 hours of liquid application. (A-B) The data presented for each condition are the mean of *n*=3 donors, and error bars represent SD. ns indicates not significant by unpaired t-test.
